# Genetic insights into psychotic major depressive disorder: bridging the mood-psychotic disorder spectrum

**DOI:** 10.1016/j.ebiom.2025.105576

**Published:** 2025-01-30

**Authors:** Thuy-Dung Nguyen, Joeri J. Meijsen, Robert Sigström, Ralf Kuja-Halkola, Ying Xiong, Arvid Harder, Kaarina Kowalec, Joëlle A. Pasman, Carolina Scarpa, Elin Hörbeck, Lina Jonsson, Sara Hägg, Niamh Mullins, Kevin S. O’Connell, Christina Dalman, Dorte Helenius, Richard Zetterberg, Henrik Larsson, Paul Lichtenstein, Ole A. Andreassen, Thomas Werge, Alfonso Buil, Mikael Landén, Patrick F. Sullivan, Yi Lu

**Affiliations:** a Department of Medical Epidemiology and Biostatistics, Karolinska Institutet, Stockholm, Sweden; b Department of Global Public Health, Karolinska Institutet, Stockholm, Sweden; c Institute of Biological Psychiatry, Mental Health Center Sct. Hans, Mental Health Services Copenhagen, Roskilde, Denmark; d Department of Psychiatry and Neurochemistry, Institute of Neuroscience and Physiology, Sahlgrenska Academy, University of Gothenburg, Gothenburg, Sweden; e Affective Clinic, Sahlgrenska University Hopsital, Gothenburg, Sweden; f College of Pharmacy, University of Manitoba, Winnipeg, Canada; g Department of Psychiatry, Amsterdam UMC Location University of Amsterdam, Amsterdam, the Netherlands; h Department of Psychiatry “ASST Fatebenefratelli-Sacco”, University of Milan, Milan, Italy; i Department of Genetics and Genomic Sciences, Icahn School of Medicine at Mount Sinai, New York, USA; j Department of Psychiatry, Icahn School of Medicine at Mount Sinai, New York, USA; k Charles Bronfman Institute for Personalized Medicine, Icahn School of Medicine at Mount Sinai, One Gustave L. Levy Pl., New York, USA; l NORMENT, Centre for Mental Disorders Research, Division of Mental Health and Addiction, Oslo University Hospital, and Institute of Clinical Medicine, University of Oslo, Oslo, Norway; m Center for Epidemiology and Community Medicine, Stockholm, Sweden; n School of Medical Sciences, Örebro University, Örebro, Sweden; o KG Jebsen Centre for Neurodevelopmental Disorders, University of Oslo and Oslo University Hospital, Oslo, Norway; p Departments of Genetics and Psychiatry, University of North Carolina at Chapel Hill, USA

**Keywords:** Psychotic major depressive disorder, Psychotic disorders, Genetic, Epidemiology

## Abstract

**Background:**

Psychotic major depressive disorder (MDD), a subtype of MDD characterised by psychotic symptoms that occur exclusively during mood episode, is clinically significant yet underexplored genetically due to its rarity. This study comprehensively examines the genetic basis of psychotic MDD and elucidates its position within the mood-psychotic spectrum.

**Methods:**

This population-based cohort study used Swedish and Danish registry data for over 5.1 M individuals born between 1958 and 1993/1996. Specialist-diagnosed psychotic MDD was defined using ICD-10 sub-codes of MDD, F32.2/F32.3. We estimated familial aggregation/coaggregation using generalised estimating equations, heritability and genetic correlations using structural equation modelling. We also analysed ∼30,000 genotyped MDD cases from the UK Biobank and a Swedish cohort to explore which polygenic risk score (PRS) may predispose individuals to psychotic MDD.

**Findings:**

With over 10,000 psychotic MDD identified from the two nationwide patient registers, this study highlights the familial aggregation of psychotic MDD, co-aggregation with mood and psychotic disorders, and its stronger genetic correlation with schizophrenia compared to non-psychotic MDD. The familial risks increased with closer biological relatedness, suggesting genetic influence. Pedigree-heritability of psychotic MDD was 30.17% (95% CI 23.53–36.80%). While the genetic correlation between psychotic and non-psychotic MDD was high (0.82, 95% CI 0.73–0.92), the psychotic subgroup showed a higher genetic correlation with schizophrenia than non-psychotic MDD (0.67 vs 0.46, p-value 7.55∗10^−4^). Within 30,000 genotyped MDD cases, individuals with psychotic MDD had higher mean PRS for schizophrenia and BD but a lower MDD PRS than non-psychotic MDD. PRS for BD type-I was associated with increased odds of psychotic MDD, while BD type-II PRS showed no significant association with psychotic MDD.

**Interpretation:**

This study provides evidence for the genetic basis of psychotic MDD, underscoring its unique position bridging the spectrum of mood and psychotic disorders. These findings advance our understanding of the aetiology of psychotic MDD and contribute to the limited body of evidence on this phenotype by utilising large-scale population-based data.

**Funding:**

10.13039/501100000781European Research Council; US National Institutes of Mental Health; European Union Horizon 2020 Program; 10.13039/501100004359Swedish Research Council; 10.13039/501100005416Research Council of Norway; Swedish Foundation for Strategic Research; 10.13039/501100003792Hjärnfonden.

**Research in context**

**Evidence before this study**

We conducted PubMed searches for papers aiming to investigate the genetic basis of psychotic major depressive disorder (MDD) using the MeSH terms “Psychotic Depression”, “Depression with Psychotic Features”. We included studies on epidemiology, genetic epidemiology, familial aggregations, and studies using genomic data.

We found that, although many studies have examined the epidemiology of psychotic MDD—with a systematic review of 99 studies up to 2018 covering incidence, prevalence among MDD cases, demographic characteristics, risk factors, and outcomes—evidence on the genetics of this phenotype is scarce and inconclusive. Previous studies, often involving fewer than 500 cases and using family designs, have yielded inconclusive findings on familial aggregation and co-aggregation. In particular, relatives of psychotic MDD probands have a higher risk of MDD and a greater prevalence of bipolar disorder compared to relatives of non-psychotic MDD probands, but findings regarding schizophrenia have been conflicting. One twin study estimated heritability based on ∼200 pairs. Recent studies using registries have suggested that psychotic MDD has a similar family genetic risk score for MDD but higher scores for bipolar disorder and schizophrenia compared to non-psychotic MDD.

Genomic studies in this area have also been limited, with most reporting null findings in candidate gene associations; however, some found associations with SNPs in genes such as brain-derived neurotrophic factor (*BDNF*), dysbindin (*DTNBP1*), dopamine beta-hydroxylase (*DBH*), and monoamine oxidase A (*MAO-A*), using samples of fewer than 400 psychotic depression cases. No genome-wide association studies (GWAS) or polygenic risk score (PRS) studies have been identified.

**Added value of this study**

This large-scale study, using nationwide Swedish and Danish registers with over 10,000 psychotic MDD cases, provides comprehensive evidence for the genetic basis of psychotic MDD. It confirms familial aggregation, heritability, and genetic overlap with mood and psychotic disorders while highlighting distinct genetic differences between psychotic and non-psychotic MDD. To our knowledge, this is the largest study to date on psychotic MDD, offering insights into its nosology and aetiology, and its unique position within the mood-psychotic disorder spectrum.

**Implications of all the available evidence**

Together with previous evidence, our findings mapped the subtypes of MDD based on psychotic features and extended evidence to the genetics of a broader mood-psychotic spectrum. We highlighted strong genetic links between psychotic MDD, SCZ, and BD type-I, while the non-psychotic form aligns more closely with MDD and BD type-II. The differential phenotypic and genetic relationships underscore the need for a comprehensive understanding of the underlying aetiology driving these complex connections. These findings have important implications for understanding its aetiology and developing targeted treatments.

## Introduction

The historical significance of the Kraepelinian dichotomy, which broadly delineates psychotic and affective illnesses, is indisputable. However, contemporary genetic research substantiates a more nuanced view of disorders along the mood-psychotic spectrum.^1^, ^2^, ^3^ Comorbidity between mood and psychotic disorders is common,^4^ and there is often diagnostic conversion from major depressive disorder (MDD) to bipolar disorder (BD)^5^^,^^6^ or schizophrenia (SCZ).^5^^,^^7^ Evidence for genetic overlap among these disorders includes familial coaggregation^8^, ^9^, ^10^ and moderate to high genetic correlations estimated from common single nucleotide polymorphism (SNPs), e.g., 0.68 for SCZ-BD,^11^ 0.44 for MDD-BD,^11^ and 0.32 for MDD-SCZ.^12^ A recent study has identified two transdiagnostic genetic liabilities underlying mood and psychotic psychopathology which are moderately correlated (0.42).^13^ Additionally, specific clinical diagnoses that feature both mood and psychotic symptoms, such as psychotic MDD, are important in elucidating the intricacies of this spectrum.

Psychotic MDD is a distinct subtype of MDD characterised by severe depressive symptoms and psychotic features, such as delusions or hallucinations, that occur exclusively during mood episodes.^14^ Despite its clinical significance, our understanding of its genetic underpinnings remains limited due to its rarity, with a lifetime prevalence of less than 1%.^15^ Previous genetic studies, often with fewer than 500 cases, have provided inconclusive findings on familial aggregation and coaggregation.^3^^,^^16^, ^17^, ^18^, ^19^, ^20^, ^21^ One twin study estimated heritability based on ∼200 pairs.^22^ Recent studies using registers have suggested that psychotic MDD has a similar family genetic risk score for MDD but higher scores for BD^23^^,^^24^ and SCZ^23^ compared to non-psychotic MDD. These findings motivated the current study to further investigate the genetic association of mood and psychotic disorders with psychotic MDD.

Leveraging nationwide registers from Sweden and Denmark with over 10,000 psychotic MDD cases, we aimed to examine the genetic basis of psychotic MDD and quantify its genetic overlap with other psychiatric disorders, particularly those within the mood-psychotic spectrum. In addition, we identified approximately 30,000 genotyped MDD cases from the UK-Biobank and a Swedish clinical cohort, further exploring the genetic risks that predispose individuals with MDD to develop psychotic MDD.

## Methods

We applied multiple statistical methods to various data sources (Table 1). We derived pedigree information in Sweden and Denmark to estimate: 1) familial aggregation of psychotic MDD, 2) familial co-aggregation with other psychiatric disorders, 3) pedigree heritability, and 4) genetic correlations with other psychiatric disorders. In the UK-Biobank and the Swedish clinical cohort with genotype data, we compared psychotic and non-psychotic MDD cases in their common variant burden using polygenic risk score (PRS).

**Table 1:** Overview of analyses.

Analyses	Estimates	Data	Methods
Familial (co)aggregation	Familial aggregation of psychotic MDD, compared to that of all MDD	Swedish + Danish register	- Full-siblings, half-siblings, cousins - Odds ratio (OR) from Generalised Estimating Equations - Robust standard error by including family as clusters - Meta-analysis
	Familial coaggregation of psychotic MDD with MDD, SCZ/SAD, Bipolar disorder, psychotic disorders-		
Heritability (*h*^*2*^)	*h*^*2*^ of psychotic MDD, compared to that of all MDD	Swedish + Danish register	- Full-siblings – cousins - Structural equation modelling - Confidence interval from Bootstrapping - Meta-analysis
Genetic correlation (*r*_*g*_)	*r*_*g*_ between psychotic MDD and non-psychotic MDD, as well as with other psychiatric traits-		
Polygenic Risk Score (PRS)	Association (OR) between PRS of MDD, SCZ, Bipolar disorders and psychotic MDD compared with non-psychotic MDD	UK-Biobank PREFECT	- Logistic regression contrasting psychotic MDD vs non-psychotic MDD - Meta-analysis

MDD: Major depressive disorder, SCZ: Schizophrenia, PRS: Polygenic risk score, OR: Odds ratio, PREFECT: Predictors for ECT.

### Data and population

#### Register data

For pedigree-based analyses, we used similar nationwide registers from Sweden and Denmark.^25^ Individuals with diagnosis of MDD and other psychiatric disorders were identified using the National Patient Registers (NPR) with records from inpatient and outpatient care. We selected similar birth cohorts, 1958–1993 in Sweden and 1958–1996 in Denmark, following them until the end of 2013 and 2016, respectively. We used the Cause of Death Register and the Total Population Register to exclude individuals who died or migrated before age 20 to ensure adequate follow-up. Siblings were identified from the Multi-Generation Register using personal identification number of biological parents. To minimise cohort effect due to variations in clinical diagnoses across birth years, we excluded sibling pairs born more than ten years apart (eMethods 1-Supplement).

#### Genotype data

For PRS analyses, we used genotype data from individuals of European ancestries in the UK-Biobank,^26^ and the PREFECT study.^27^ The UK-Biobank recruited over 500,000 adults aged 37–73 from the UK. Genotype data were available after quality control and imputation.^26^ The PREFECT study included 2904 individuals who received electroconvulsive therapy (ECT) and were registered in the Swedish National Quality Register for ECT during 2013–2017.^27^ DNA samples were extracted from blood and genotyped using Illumina GSA-MD array (v1). Quality control and imputation protocols were provided elsewhere^27^ (eMethods 2).

### Phenotypes

We used ICD codes to define psychiatric disorders (eTables S1 and S2). **MDD** cases were defined as those receiving any MDD diagnosis from inpatient or outpatient care in the registers and PREFECT data. In UK-Biobank data, MDD was defined by diagnoses recorded in the inpatient hospital records. Among MDD cases, we identified **psychotic MDD** as having at least one diagnosis of psychotic MDD (ICD-10 codes F32.3 or F33.3). We did not exclude specialist diagnoses of BD, SCZ, or schizoaffective disorder (SAD), as such exclusions would limit the possibility for investigating diagnostic conversion as part of the phenotype characterization. **Non-psychotic MDD** included individuals without any psychotic MDD diagnosis.

As sensitivity analyses, we derived a restrictive definition of psychotic MDD, same as a previous study^24^ to minimise the impact of BD, SCZ, or SAD, by excluding BD from all MDD cases and further excluding SCZ and SAD from psychotic MDD cases.

### Statistical analyses

We examined psychotic MDD and its relationship with mood-psychotic disorders in the whole population by describing the percentage of individuals with diagnosis of SCZ/SAD, BD, or other non-affective psychotic disorders (i.e., ICD-10 codes F2 excluding F20, F25 and equivalent ICD-8/9 codes), among both psychotic and non-psychotic MDD groups. The proportion who received antipsychotic (ATC code N05A) and ECT treatments (procedure codes 9218, V9218, DA006, DA024, DA025) in each group were also described.

#### Familial aggregation and co-aggregation

We studied the familial aggregation of psychotic MDD and its co-aggregation with MDD, SCZ/SAD, BD, and all psychotic disorders. For each type of relative (full-siblings, half-siblings, and cousins), we calculated odds ratios (OR) for a relative having psychotic MDD (aggregation) or other disorders (co-aggregation) if the proband has psychotic MDD compared with a proband without. Sex- and birth-year-adjusted ORs were estimated using Generalised Estimating Equation with a logit link function (*gee()* function in the R *drgee* package). To account for non-independency between observations due to the inclusion of all relative pairs, robust standard errors (SE) were estimated by including family as clusters in the models (eMethods 3). To correct for multiple testing, we used the Bonferroni correction by adjusting the p-value threshold to 0.05/number of tests.

#### Pedigree heritability (*h*^*2*^) and genetic correlation (r_g_)

We applied structural equation models (R *OpenMx* 2.19.8) to estimate heritability (*h*^*2*^) on liability-scale) and genetic correlation (*r*_*g*_). To maximise the number of concordant case pairs, we contrasted full-siblings and cousins, the majority of relative pairs in our populations. Variance and covariance matrices were used to estimate additive genetic (*A*), shared environment (*C*), and unique environment (*E*). All models relied on the assumption of no interaction between genetic and environmental effects. Full-siblings were assumed to share 50% of *A* and 100% of *C*, while cousins share 12.5% of A and no *C*. SE were estimated using Bootstrap resampling of families (1000 replicates). For *h*^*2*^, we fitted ACE and AE models using weighted least squares (WLS) and presented AE models as the main results. For *r*_*g*_ of non-mutually exclusive traits (i.e., one person can have both traits), we fitted similar ACE and AE models and compared model fit using a Chi-squared test. For *r*_*g*_ of mutually exclusive traits (e.g., psychotic and non-psychotic MDD), we used Maximum Likelihood (ML) estimators and compared model fit using a likelihood ratio test (eMethods 4).

#### PRS

We computed PRS of MDD, SCZ, BD, BD type-I and type-II in the UK-Biobank and PREFECT samples using the latest genome-wide association studies (GWAS) summary statistics. To avoid potential sample overlap between discovery GWAS and target samples, we excluded the relevant population in the discovery GWAS (eTable S3). We further filtered duplicate SNPs, rare variants (MAF < 0.01), low-quality imputed SNPs (INFO score < 0.9 if available), and removed the major histocompatibility complex regions (chr6:28–34 Mb). We used SBayesR in GCTB to rescale SNP effects while accounting for linkage disequilibrium, followed by PRS calculation using PLINK 2.0. Each PRS were standardised before the analyses. In logistic regression models, we estimated the OR associated with per standard deviation (SD) increase in PRS, adjusted for sex, age at data collection and the first five genetic principal components. We ran both univariate (i.e., testing each PRS separately) and multivariable models (i.e., PRS mutually adjusted in a single model) (eMethods 5).

#### Meta-analysis

Estimates were obtained from each dataset and then meta-analysed using fixed-effect models (eMethods 6). Meta-analysis results were reported as primary findings in the main text.

### Sensitivity analysis

We conducted several sensitivity analyses using the restrictive definitions of psychotic MDD (see *Phenotypes*), including the estimations of heritability and genetic correlations in Swedish sample, and PRS association in all genotyped samples. Furthermore, we conducted a sensitivity analysis in the UK-Biobank on severe MDD cases only (i.e., comparing psychotic MDD [F32.3/F33.3] with *severe* non-psychotic MDD [F32.2/F33.2]).

### Ethics

This study was approved by the Swedish Ethical Review Authority (2013/862-31/5, 2016/1214-32) and the Regional Ethics Review Board in Stockholm (2012/1969-31/1). Danish data use was authorised by the Danish Health Data Authority (FSEID-00003339) and the Danish Data Protection Agency. PREFECT study was approved by the Regional Ethics Review Board in Stockholm (2012/1969-31/1). UK-Biobank data use was approved via application 22224.

### Role of funders

Funders were not involved in the study design, data collection, data analysis, data interpretation, or manuscript writing.

## Results

We included over 5.1 million individuals from Sweden and Denmark (Table 2), with 49% females and age ranges of 20–59 years at the end of follow-up. The prevalence of specialist-treated MDD was 5.5% in Sweden and 4.5% in Denmark. We identified 10,172 psychotic MDD cases (3.3% of MDD cases identified in Sweden and 5.2% in Denmark), with a population prevalence of ∼0.2% in both countries (Table 2, eTable S4a). In the genotyped cohorts of PREFECT and the UK-Biobank, we identified a total of 29,285 MDD and 1288 psychotic MDD. The proportion of psychotic MDD was much higher in PREFECT because psychotic MDD is an indication for receiving ECT.

**Table 2:** Sample size and population characteristics.

	Swedish register	Danish register	PREFECT	UK-Biobank
Total included	3,171,281	1,947,391	2892 ECT-treated MDD	26,393 MDD
Female (%)	1,540,294 (48.57%)	953,276 (48.95%)	1802 (62.31%)	16691 (63.24%)
Age at end of follow-up	Mean 37.44, SD 9.86, range 21–55	Mean 39.71, SD 11.15, range 20–59	Mean 53.73, SD 16.34	Mean 56.63, SD 8.04
MDD (ICD 8,9,10) (% in study population)	173,742 (5.48%)	88,497 (4.54%)	2,892^b^	26,393^b^
MDD (ICD 10) (% in study population)	168,186 (5.30%)	87,940 (4.52%)		
Psychotic MDD (% in all MDD^a^)	5597 (3.33%)	4575 (5.20%)	746 (25.80%)	542 (2.05%)
Psychotic MDD (% in study population)	5597 (0.18%)	4575 (0.23%)	–	–

MDD: Major depressive disorder, SD: Standard deviation, PREFECT: Predictors for ECT, ECT: Electroconvulsive therapy, ICD: International classification of diseases.

a Proportion among all MDD ICD 10 cases.

b Defined using only ICD 10 codes.

**Fig. 1:**
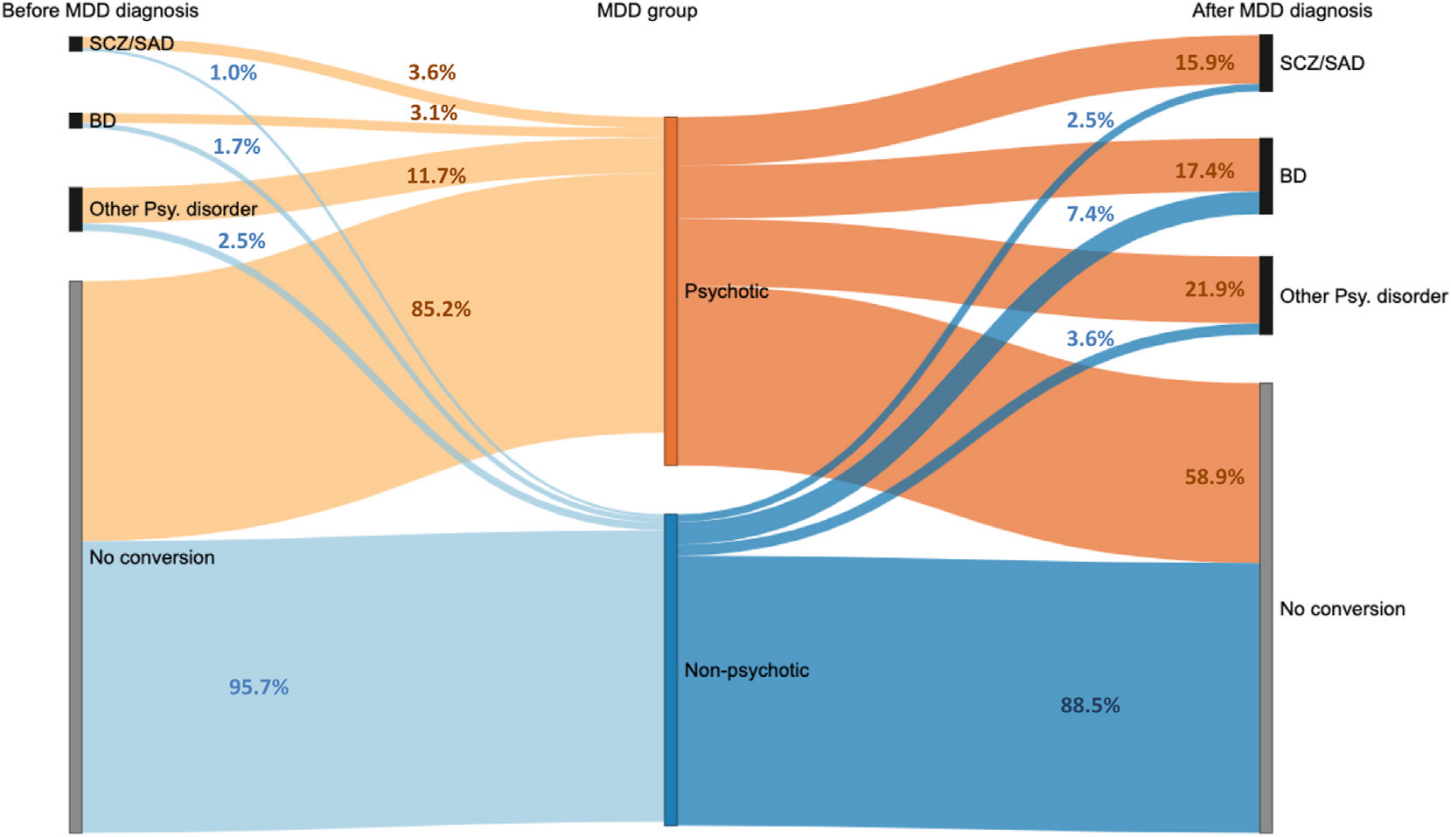
**Conversion between psychotic/non-psychotic major depressive disorder (MDD) and other psychotic disorders.** The Sankey diagram shows the proportions of individuals diagnosed with bipolar disorders, schizophrenia/schizoaffective disorder and other psychotic disorders (not including BD/SCZ/SAD) before and after the first MDD diagnosis, separately for psychotic and non-psychotic MDD. The width of the links represents the proportion of individuals with these psychotic disorders in psychotic/non-psychotic MDD group. Groups are non-mutually exclusive. BD: Bipolar disorder, SCZ/SAD: Schizophrenia/Schizoaffective disorder, Psy. Disorder: Psychotic disorder.

We examined the phenotype of psychotic MDD in the Swedish and Danish populations. Most cases had no lifetime diagnosis of SCZ/SAD, BD, or other psychotic disorders. A small proportion of cases had these other diagnoses before the first MDD diagnosis. Compared to non-psychotic MDD, we found higher proportions of psychotic MDD converted to SCZ/SAD (15.9%), BD (17.4%), and other psychotic disorders (21.9%) (Fig. 1, eTable S4b). In terms of treatment (Swedish data only), individuals with psychotic MDD were three times more likely to be prescribed antipsychotic medications than those with non-psychotic MDD (73.86% compared to 23.92%), and a significantly higher percentage received ECT (14.65% compared to 1.86%).

### Psychotic MDD aggregates within family and co-aggregates with other psychiatric traits

**Fig. 2:**
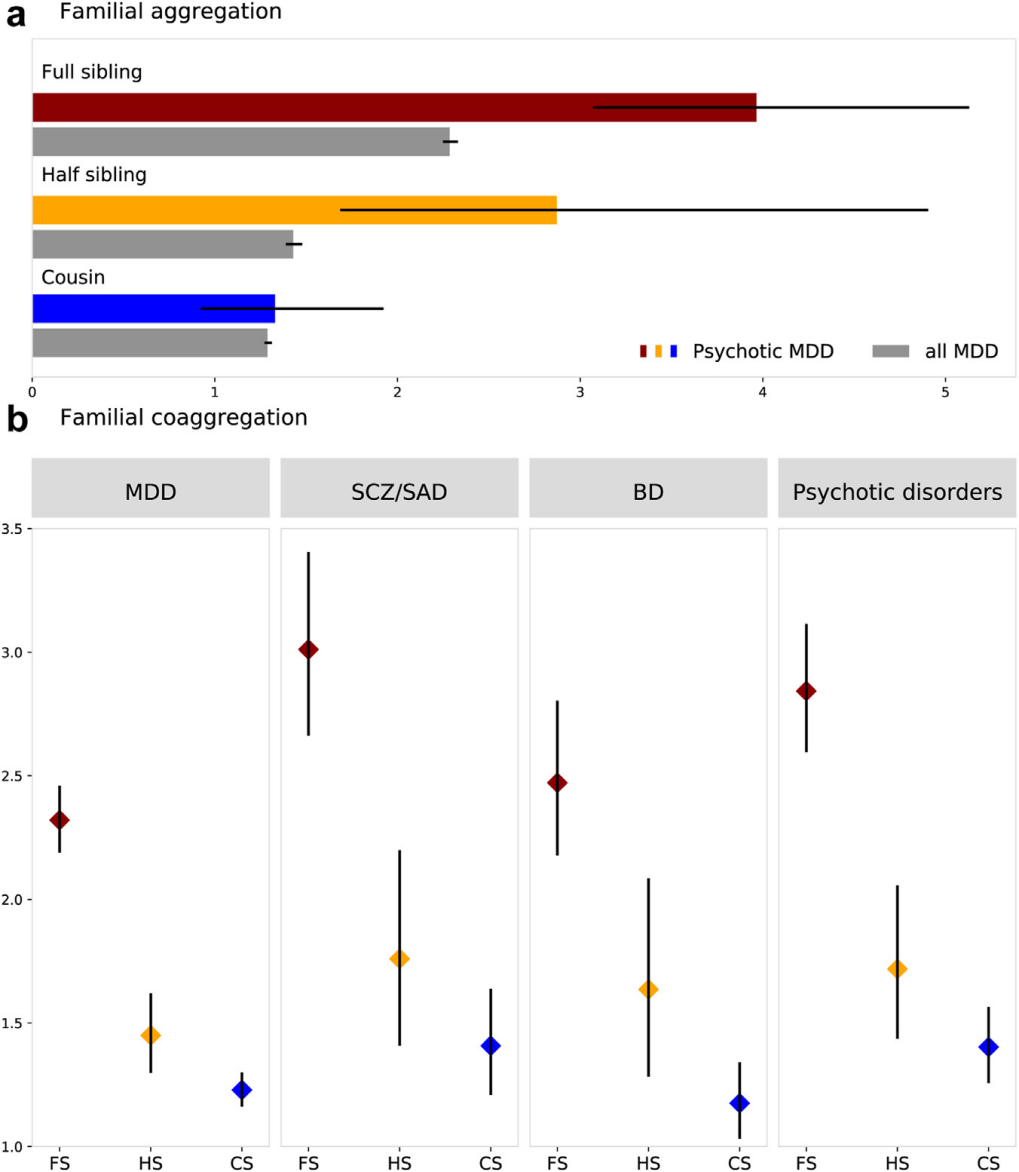
**Familial risk. a. Familial aggregation** of psychotic MDD, and all MDD; **b. Familial coaggregation** of psychotic MDD with MDD, schizophrenia/schizoaffective disorder, bipolar disorder and all psychotic disorders within family separately for full-siblings, half-siblings, and cousins. Bars and dots show OR, error bars show 95% CI for the estimates. Colours represent types of siblings. Combined estimates from meta-analyses of Sweden and Denmark. MDD: Major depressive disorder, SCZ/SAD: Schizophrenia/Schizoaffective disorder, BD: Bipolar disorder, FS: Full-siblings, HS: Half-siblings, CS: Cousins.

We extracted 2,574,835 full sibling pairs, 512,084 half-sibling pairs and 6,326,866 cousin pairs (number of concordant/discordant pairs in eTable S5). Analyses were conducted separately for Swedish and Danish data, yielded generally comparable estimates (eTables S6 and S7). Psychotic MDD aggregated within families, such that relatives of individuals with psychotic MDD had elevated risks for the same condition. The familial risk increased with closer genetic relationships: OR 1.33 (95% CI = 0.92–1.92) for cousins, 2.88 (95% CI = 1.69–4.90) for half-siblings, and 3.97 (95% CI = 3.07–5.13) for full-siblings, suggesting a genetic influence. For full- and half-siblings, ORs for psychotic MDD were nearly twice as high as those for all MDD (p-value < 0.017 after Bonferroni correction) (Fig. 2a; eTable S6).

We observed familial coaggregation between psychotic MDD and other psychiatric disorders including MDD, SCZ/SAD, BD and all psychotic disorders. Relatives of individuals with psychotic MDD had significantly increased risks for all studied disorders, with risks increasing with closer genetic relationships (OR ranged between 1.18 and 1.41 for cousins, 1.45–1.76 for half-siblings, and 2.32–3.01 for full siblings), also suggesting genetic influences on these coaggregations (Fig. 2b, eTable S7).

### Psychotic MDD has high genetic correlation with schizophrenia or schizoaffective disorder

We estimated heritability and genetic correlations by contrasting full-siblings and cousins (intraclass correlations in eTable S8). The common environment component (C) estimated from ACE models were not significantly different from null in both Swedish (0.02%, p-value = 0.998 for testing C = 0) and Danish (3.59%, p-value = 0.824 for testing *C* = 0) data. The *h*^*2*^ for psychotic MDD from the AE model was 30.17% (95% CI 23.53–36.80%), lower than that of all MDD (40.05%, 95% CI 39.12–40.99%); however, heterogeneity was observed in country-specific estimates (eTables S9 and S10). In sensitivity analyses, we found a lower *h*^*2*^ for the restrictive definition (22.92%, 95% CI 7.53–35.58%). The confidence interval overlapped with the main definition but wider due to fewer concordant case pairs observed (eTables S16 and S17).

**Fig. 3:**
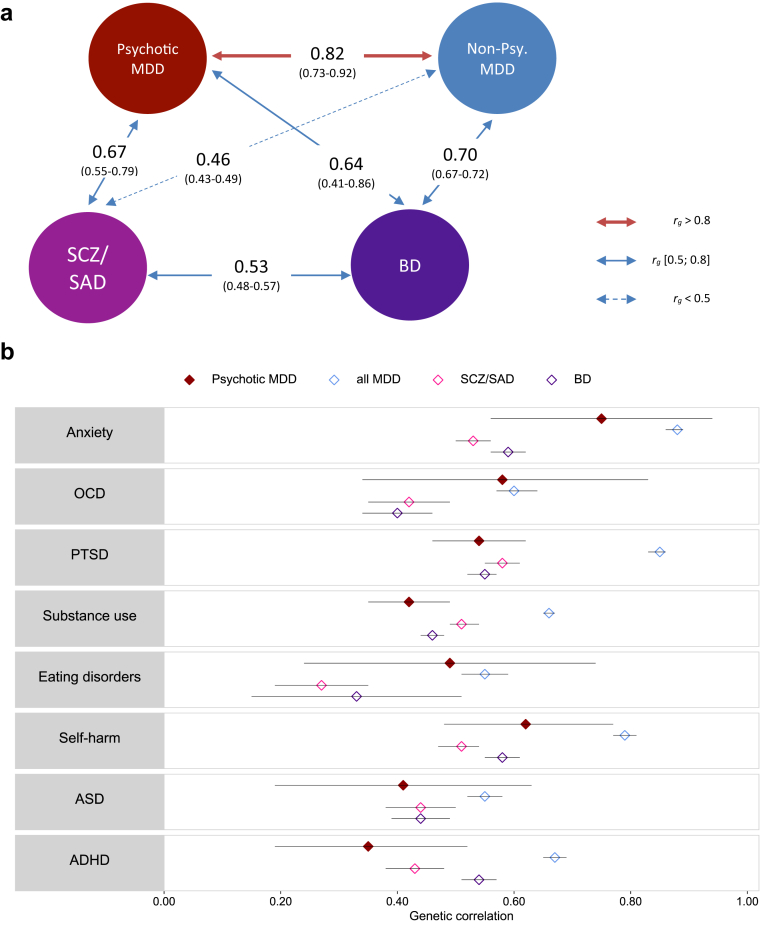
**Genetic correlation between psychotic MDD and other psychiatric traits. a.** Genetic correlation between psychotic and non-psychotic MDD, and their correlations with schizophrenia & bipolar disorder. *R*_*g*_ with schizophrenia/schizoaffective disorder and bipolar disorder. Colour and width of arrows represent magnitude of the estimates. **b.** Genetic correlation with other psychiatric traits. *R*_*g*_ with other psychiatric traits. Coloured shapes represent point estimates, error bars represent 95% CI. Estimates from AE models. 95% CI from Bootstrap resampling analyses (1000 replicates). MDD: Major depressive disorder, SCZ/SAD: Schizophrenia/Schizoaffective disorder, BD: Bipolar disorder, OCD: Obsessive-Compulsive Disorder, PTSD: Post-Traumatic Stress Disorder, ASD: Autism Spectrum Disorder, ADHD: Attention-Deficit/Hyperactivity Disorder.

To evaluate *r*_*g*_ among disorders within the mood-psychotic spectrum, we estimated the *r*_*g*_ between psychotic and non-psychotic MDD, and their respective *r*_*g*_ with SCZ/SAD and BD. We benchmarked these estimates against the known *r*_*g*_ between SCZ/SAD and BD (0.53). The *r*_*g*_ between psychotic and non-psychotic MDD was high but different from one (0.82, 95% CI 0.73–0.92, p-value = 1.66∗10^−4^ for testing *r*_*g*_ = 1). Psychotic MDD had moderate to high *r*_*g*_ with SCZ/SAD (0.67, 95% CI 0.55–0.79) and BD (0.64, 95% CI 0.41–0.86). Compared to non-psychotic MDD, the *r*_*g*_ with SCZ/SAD was significantly higher for psychotic MDD (0.67 vs 0.46, p-value = 7.55∗10^−4^), whereas the *r*_*g*_ with BD was more similar (0.64 vs 0.70, p-value = 0.06) (Fig. 3a, eTables S11 and S13). These results were similar in the restrictive definition excluding BD and SCZ/SAD from psychotic MDD (eTable S18). For instance, the *r*_*g*_ with SCZ/SAD was 0.62 and 0.39 for psychotic and non-psychotic MDD, respectively. The *r*_*g*_ with BD was lower in the restrictive definition of psychotic MDD albeit with a large confidence interval (0.37, 95% CI = 0.14–1.00).

Next, we estimated the *r*_*g*_ of psychotic MDD with other psychiatric disorders, and compared them to the corresponding *r*_*g*_ of all MDD (>95% were non-psychotic), SCZ/SAD and BD. Psychotic MDD had moderate to high *r*_*g*_ with other psychiatric disorders (range 0.35–0.75). Compared with all MDD, psychotic MDD showed lower *r*_*g*_ with other psychiatric disorders, though CIs overlapped for many disorders. Notably, the *r*_*g*_ of psychotic MDD with PTSD, substance use disorders and ADHD were significantly lower than the corresponding estimates of all MDD, but more similar to the estimates of SCZ/SAD and BD (Fig. 3b, eTables S12 and S13).

### Higher PRS burden of SCZ and BD type-I among psychotic MDD compared to non-psychotic MDD

We further explored the question that within MDD cases, which PRS predispose individuals to develop psychotic MDD. To address that, we derived PRS for PREFECT and UK-Biobank samples using the latest GWAS of MDD, SCZ, and BD (type-I, type-II, and both). We compared these PRS between MDD cases with and without psychotic features (i.e., case–case comparison).

In univariate models, higher PRS for these psychiatric disorders were associated with increased odds of psychotic MDD, except for the PRS of MDD and BD type-II (eTable S14a). The highest OR was for BD type-I (OR = 1.33, 95% CI 1.25–1.41), followed by SCZ (OR = 1.30, 95% CI 1.23–1.38) and all BD (OR = 1.28, 95% CI 1.20–1.36). Intriguingly, the PRS of MDD was associated with lower odds of psychotic MDD (OR = 0.93, 95% CI 0.88–0.99).

**Fig. 4:**
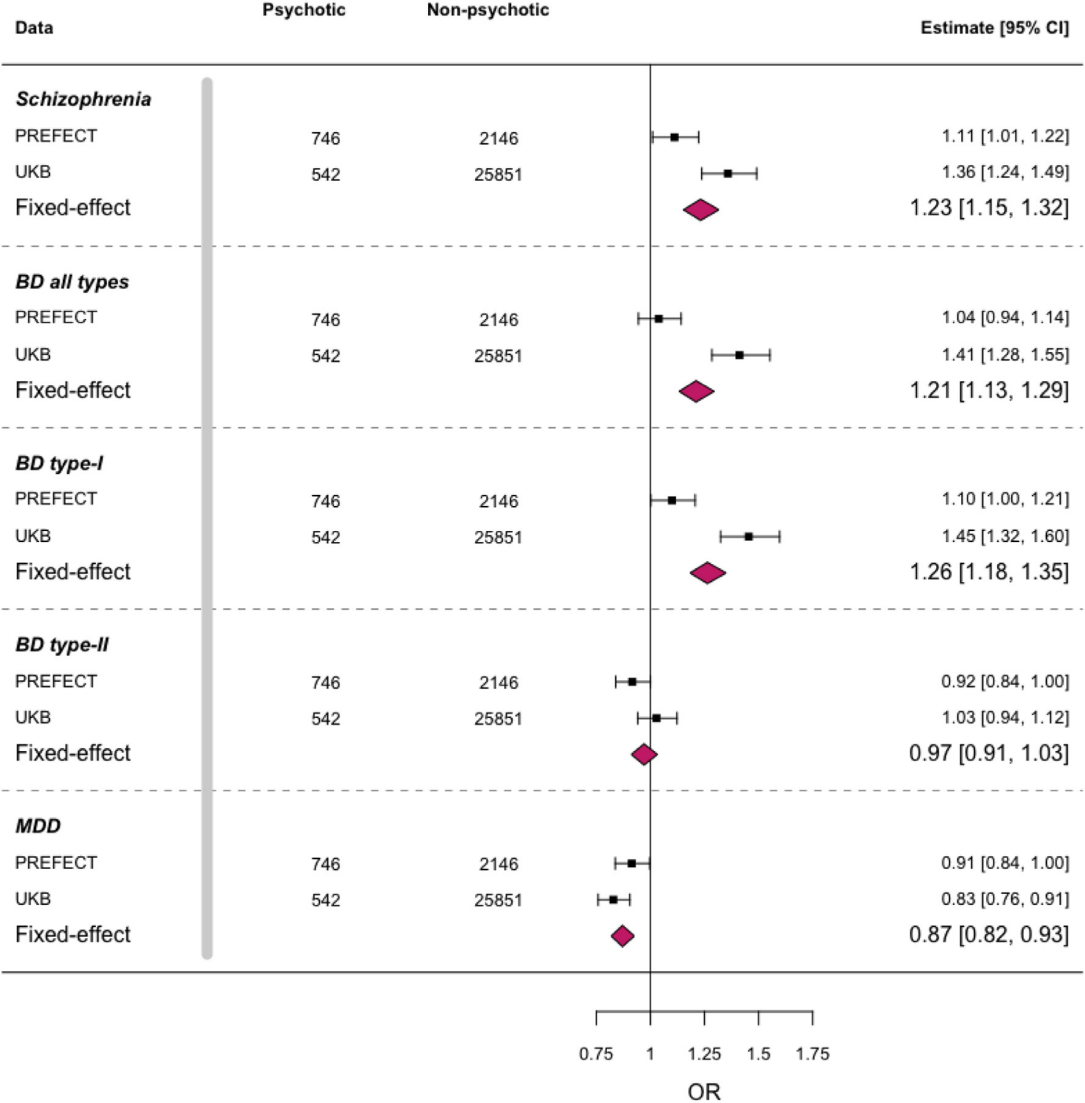
**Regression analyses investigating the association between various polygenic risk scores and the occurrence of psychotic MDD.** Forest plots display the association between PRS of schizophrenia, bipolar disorders, MDD and psychotic MDD. Results are from joint models including PRS of multiple disorders and covariates. Black squares and error bars represent the odds ratio and 95% CI for having psychotic MDD instead of non-psychotic MDD associated with a 1-SD-increase in PRS, shown separately for PREFECT and UK-Biobank data. The coloured diamonds show combined estimates. MDD: Major depressive disorder, BD: Bipolar disorder.

Given *r*_*g*_ among these disorders, we then tested PRS in a joint model. Similar to the univariate results, individuals with psychotic MDD had higher PRS of SCZ and BD compared to non-psychotic MDD. Specifically, each SD increase in SCZ or BD PRS was associated with 21–23% higher odds of having psychotic MDD. For BD subtypes, BD type-I PRS was associated with higher odds of psychotic MDD (OR = 1.26, 95% CI = 1.18–1.35), while BD type-II PRS was not significantly associated (OR = 0.97, 95% CI = 0.91–1.03) (Fig. 4, eTable S14a).

Adjusting for the PRS of SCZ and BD, individuals with psychotic MDD had lower MDD PRS compared to non-psychotic MDD. Each SD increase in MDD PRS associated with lower odds of psychotic MDD (OR = 0.87, 95% CI = 0.82–0.93) (Fig. 4). Sensitivity analyses showed similar results for the restrictive definition of psychotic MDD (eTable S19a). Furthermore, comparing psychotic MDD with severe non-psychotic MDD, OR for all PRS were only slightly attenuated, suggesting that the observed genetic associations are not solely attributed to differences in severity among psychotic and non-psychotic MDD (eTables S14b and S19b).

## Discussion

This study aimed to elucidate the genetic basis of psychotic MDD and its position within the mood-psychotic disorder spectrum. We found that psychotic MDD aggregated within families and co-aggregated with mood and psychotic disorders. The heritability estimate was 30.2%, and it showed moderate to high genetic correlations (0.35–0.75) with other psychiatric disorders, including SCZ/SAD, BD, anxiety, OCD, PTSD, substance use disorders, eating disorders, intentional self-harm, ASD, and ADHD. Within MDD cases, psychotic MDD had higher PRS for SCZ and BD type-I than non-psychotic MDD, but had similar PRS for BD type-II and lower PRS for MDD. These findings highlight the unique position of psychotic MDD within the mood-psychotic genetic spectrum, as its genetic liability is markedly influenced by SCZ, BD type-I, and MDD.

First, using nationwide registers from Sweden and Denmark, we characterised psychotic MDD at the population level, with a prevalence of 0.2% observed in both countries. Approximately 3–5% of specialist-treated MDD cases had psychotic feature, consistent with a previous report (5%) in outpatient settings.^28^ Compare to non-psychotic MDD, psychotic MDD showed much stronger phenotypic associations with BD, SCZ/SAD, or other psychotic disorders, which aligned with earlier studies that showed individuals with psychotic MDD are at a higher risk for developing bipolar disorder compared to those with non-psychotic MDD.^29^^,^^30^ These data underscore the need for a comprehensive understanding of the underlying aetiology driving these complex relationships.

Second, our study confirms that psychotic MDD is heritable, with a heritability estimate similar to what previously reported, 39% (95% CI 20–56%).^22^ The slightly lower estimate was likely due to differences in study population, sample size, and phenotype ascertainment. While the previous study included ∼200 twin pairs of male veterans with self-reported MDD, our study involved over 10,000 specialist-treated psychotic MDD cases from two Nordic countries, providing a more precise estimate (*h*^*2*^ = 30.2%, 95% CI = 23.5–36.8%). Despite higher familial aggregation of psychotic MDD compared to all MDD, we did not find a higher heritability for the psychotic subgroup (though the two measures are on different scales, thus estimates are not directly comparable). This was unexpected given previous research showing higher heritability for more severe manifestations of MDD.^31^^,^^32^ We note regional differences influencing phenotype quality, such as sub-code utilization, may have affected results. For example, the heritability of psychotic MDD in Sweden (20%) was considerably lower than in Denmark (37%), while our previous estimates for all MDD were similar between the two countries.^33^

Third, psychotic MDD, as a subtype of MDD, had partially distinct genetic makeups from the non-psychotic form. The high genetic correlation (*r*_*g*_ = 0.82) between psychotic and non-psychotic MDD aligns with findings for other MDD subgroups^31^^,^^32^ and BD subtypes.^11^ Moreover, genetic correlations with other psychiatric disorders were generally lower for psychotic MDD compared to all MDD, particularly with PTSD, substance use disorders and ADHD. Both psychotic and non-psychotic subtypes showed a higher PRS for MDD compared to those without MDD (eTable S15), but the MDD PRS was associated with a lower OR for the psychotic subtype, suggesting a lower genetic burden of MDD compared to non-psychotic MDD.

Finally, psychotic MDD shows stronger genetic correlation with SCZ/SAD and a higher PRS burden for BD type-I compared to non-psychotic MDD. Psychotic MDD may be expected to exhibit a larger genetic overlap with psychotic disorders like SCZ than other MDD subtypes,^3^^,^^23^ but the precise extent of overlap was unknown. Our study, showed a substantial genetic correlation (*r*_*g*_ = 0.67) between psychotic MDD and SCZ/SAD, with each SD increase in SCZ PRS associated with a 23–30% increased risk for psychotic MDD compared to the non-psychotic form. Regarding BD, we observed an equally high genetic correlation with both psychotic and non-psychotic MDD, but these overlaps varied by BD subtypes. Specifically, psychotic MDD was significantly associated with PRS of BD type-I, not type-II, suggesting a closer relationship with BD type-I. Previous research has demonstrated the genetics of mood disorder spectrum, with schizoaffective disorder and BD type-I on one end, depressive disorders on the other end, and BD type-II in an intermediate position.^34^ Our findings mapped the subtypes of MDD based on psychotic features and extended evidence to the genetics of a broader mood-psychotic spectrum. We highlighted strong genetic links between psychotic MDD, SCZ, and BD type-I, while the non-psychotic form aligns more closely with MDD and BD type-II.

Psychotic MDD has been understudied despite its clinical significance. We used nationwide registers and genotype data which allowed a comprehensive genetic investigation. However, using ICD-10 sub-codes in the registers to define the phenotype has not undergone validation. We, therefore, performed analyses using two definitions of psychotic MDD. The consistent results across these definitions reinforce the validity of our conclusions. To our knowledge, our study is the most extensive to date on the genetic basis of psychotic MDD. Nevertheless, some limitations remain. The sample size of psychotic MDD, especially concordant case pairs, was relatively small compared to other psychiatric disorders. Combining data from Sweden and Denmark with up to 40 years of follow-up partially mitigated this but resulted in heterogenous heritability estimates, possibly due to differences in healthcare organisation or clinical culture.^33^ Additionally, the accuracy of patient-reported symptoms (occasional or subtle delusions or hallucinations may be underreported) and clinician records may vary across data sources, and we do not have access to the symptom-level data. Besides, we did not explore gene-environment interactions in this study. Our heritability and genetic correlation models assumed no such interaction, which, if violated, could impact the estimates. Finally, our PRS analyses were limited to individuals of European ancestry and had differential power due to different GWAS sample sizes (e.g., smaller BD type-II GWAS than BD type-I). Future research in other ancestry^35^ and using larger GWAS training sets is warranted.

In summary, our study provides comprehensive evidence for the genetic basis of psychotic MDD, highlighting its unique position bridging mood and psychotic disorders. These findings have important implications for understanding its aetiology and developing targeted treatment. Using extensive population-based data and genetic analyses, our study significantly contributes to the limited research on psychotic MDD and advances our understanding of the genetic heterogeneity of MDD.^36^, ^37^, ^38^

## Contributors

YL conceived the idea, designed and supervised the implementation of the project. TDN designed the study, performed analyses using Swedish data, conducted meta-analyses of Swedish and Danish results, interpreted the results, drafted and revised the manuscript. JJM contributed to the study design, performed analyses using Danish data, and interpreted the results. RKH contributed to the study design, supervised the statistical analyses, and interpreted the results. YX, AH conducted the PRS analyses. RS, KK, JAP, CS, EH, LJ, CD, DH, RZ, PFS contributed to the discussion on methodological issues, and interpretation of the results. SH provided access to the UK-Biobank data (application Number 22224) and interpretation of the results. NM, KSO, OAA contributed GWAS summary statistic data and interpretation of the results. HL, PL provided access to the Swedish register data. TW, AB provided access to the Danish register data, contributed to the study design, and interpretation of the results. ML provided access to the PREFECT data, and interpretation of the results. All authors discussed and commented on the manuscript. All authors read and approved the final version of the manuscript.

TDN had full access to all the Swedish register, PREFECT and UK-Biobank data used in the study and takes responsibility for the integrity of the data and the accuracy of the data analysis. JJM had full access to all the Danish register and takes responsibility for the integrity of the data and the accuracy of the data analysis.

TDN and JJM share first authorship.

## Data sharing statement

The raw data are protected and are not available for sharing due to data privacy laws. For the Danish register data, only Danish research environments are granted authorization to Danish data. Foreign researchers can, however, get access to data. Further information on data access can be found at https://www.dst.dk/en/TilSalg/Forskningsservice, or by contacting JJM (joeri.jeroen.meijsen@regionh.dk). For the Swedish register data, researchers can apply for data access at Statistics Sweden (SCB, https://www.scb.se/en/) and The National Board of Health and Welfare (Socialstyrelsen, https://www.socialstyrelsen.se/). UK-biobank data is accessible via application at https://www.ukbiobank.ac.uk/enable-your-research/apply-for-access. Questions about Swedish register and PRECT study data should be sent to the corresponding author.

## Declaration of interests

PFS was a paid advisor and shareholder for Neumora Therapeutics. ML has received lecture honoraria from Lundbeck pharmaceuticals outside the submitted work. OA is a consultant to Cortechs.ai and Precision-Health.AI and has received speaker’s honorarium from Otsuka, Lundbeck, Janssen and Sunovion. HL has received grants from Shire/Takeda, lecture honoraria from Shire/Takeda, Medici and Elovan, all outside the submitted work Coauthors declare no conflict of interest.

## Appendix ASupplementary data

Supplementary methods and eTables S1–S19
