# Supplementary material for: Genetic insights into psychotic major depressive disorder: bridging the mood-psychotic disorder spectrum
Source: eBioMedicine. 2025 Jan 30;112:105576. doi: 10.1016/j.ebiom.2025.105576 (PMC11830301; doi:10.1016/j.ebiom.2025.105576)
Supplement: Supplementary methods and eTables S1–S19 [file mmc1.docx]

Data supplement for:

**Genetic Insights into Psychotic Major Depressive Disorder:**

**Bridging the Mood-Psychotic Disorder Spectrum**

Thuy-Dung Nguyen, Joeri J. Meijsen, Robert Sigström, Ralf Kuja-Halkola, Ying Xiong, Arvid Harder, Kaarina Kowalec, Joëlle A. Pasman, Carolina Scarpa, Elin Hörbeck, Lina Jonsson, Sara Hägg, Niamh Mullins, Kevin S. O'Connell, Christina Dalman, Dorte Helenius, Richard Zetterberg, Henrik Larsson, Paul Lichtenstein, Ole A. Andreassen, Thomas Werge, Alfonso Buil, Mikael Landén, Patrick F. Sullivan, Yi Lu

Table of Contents

[I. Supplementary methods 2](#_Toc185836104)

[eMethods 1. Register data 2](#_Toc185836105)

[eMethods 2. Genotype data 3](#_Toc185836106)

[eMethods 3. Estimate familial aggregation and coaggregation 4](#_Toc185836107)

[eMethods 4. Pedigree heritability and genetic correlation 4](#_Toc185836108)

[eMethods 5. Computing PRS 5](#_Toc185836109)

[eMethods 6. Meta-analyses 6](#_Toc185836110)

[II. Supplementary tables 7](#_Toc185836111)

[eTable 1: Phenotype definitions in each dataset 7](#_Toc185836112)

[eTable 2: ICD codes 8](#_Toc185836113)

[eTable 3: Summary statistics used for PRS analyses 13](#_Toc185836114)

[eTable 4: Proportion of psychotic MDD and conversion rate to other psychotic disorders 14](#_Toc185836115)

[eTable 5: Number of concordant/discordant pairs of MDD phenotypes in register data 15](#_Toc185836116)

[eTable 6: Familial aggregation separately for Sweden and Denmark 16](#_Toc185836117)

[eTable 7: Familial coaggregation separately for Sweden and Denmark 17](#_Toc185836118)

[eTable 8: Intraclass correlation for MDD phenotypes in register data 18](#_Toc185836119)

[eTable 9: Heritability estimates separately for Sweden and Denmark 19](#_Toc185836120)

[eTable 10: Hypothesis test for difference in heritability estimates 20](#_Toc185836121)

[eTable 11: Genetic correlation between psychotic subgroups of MDD and SCZ/SAD, BD 20](#_Toc185836122)

[eTable 12: Genetic correlation between psychotic MDD, MDD, SCZ/SAD, BD and psychiatric disorders 21](#_Toc185836123)

[eTable 13: Hypothesis testing for difference in genetic correlations 23](#_Toc185836124)

[eTable 14: PRS regression models separately for UKB and PREFECT 24](#_Toc185836125)

[eTable 15: Logistic regression comparing MDD PRS between psychotic/non-psychotic MDD with non-MDD 25](#_Toc185836126)

[III. Supplementary results from sensitivity analyses 26](#_Toc185836127)

[eTable 16: Number of cases, concordant/discordant pairs, intraclass correlations for restrictive psychotic MDD 26](#_Toc185836128)

[eTable 17: Heritability estimates for restrictive definition of psychotic MDD 26](#_Toc185836129)

[eTable 18: Genetic correlation between psychotic subgroups of MDD based on restrictive definition and SCZ/SAD, BD 26](#_Toc185836130)

[eTable 19: PRS regression models separately for UKB and PREFECT for restrictive psychotic MDD 27](#_Toc185836131)

[References 28](#_Toc185836132)

1. **Supplementary methods**

## **eMethods 1. Register data**

For the pedigree-based analyses, we used several population-based registers in Sweden and Denmark. Individual information from different registers were linked together via the unique personal identification numbers given at birth or migration. We selected similar birth cohorts, 1958-1993 in Sweden and 1958-1996 in Denmark, and followed until the end of 2013 and 2016 in the two countries, respectively. Briefly, individuals with MDD and other psychiatric disorders were identified using the National Patient Registers, which include records from inpatient (started in 1973 in Sweden; and 1969 in Denmark) and outpatient care (started in 2001 in Sweden; and 1995 in Denmark).^1,2^ We used the Cause of Death Register^3,4^ and the Total Population Register^5,6^ to exclude individuals who died or migrated before the age 20 to ensure adequate follow-up time. Siblings were identified from the Multi-Generation Register using personal identification number of biological parents.^7,8^ To minimize impact of birth cohort (e.g., due to changing clinical practice) between siblings born in different year, we excluded sibling pairs born more than ten years apart. Details about each registry in each country are presented below:

Population register:

The national population registers are mandatory registration that contain information about birth, death, migration and marital status of individual who were born or migrated to Sweden and Denmark.^5,6^

The Swedish Total Population Register (TPR) started in 1968 which records everyone who are born in or moved to Sweden. The coverage of the register is high with 99.9% of all births and more than 90% of migrations registered.^5^

In Denmark, the Danish Civil Registration System (CRS) was established in 1968. The register includes livebirths to registered mothers, individuals who registered in a Danish electronic church register and legal residents in Denmark for at least 3 months. The register also includes residents of Greenland (an autonomous country within Denmark) since 1972.^6^

Patient registers:

In Sweden, the National Patient Register includes data from the inpatient and outpatient specialist care.^1^ The register started in 1964 with somatic diseases, then added psychiatric diagnosis from 1973. In 2001, diagnoses that were given by specialist in outpatient care were also included. Diagnoses were recorded in the register using ICD codes. The ICD versions used includes ICD 6 from 1964-1976, ICD 7 from 1967-1987, ICD 9 from 1987 to 1996, ICD 10 from 1997 onward. The coverage of the inpatient register improved over time with around 85% of somatic care covered in 1983, and with almost 100% coverage somatic and psychiatric care in 1987. The coverage of the outpatient care is about 80% with the majority missing data is from private caregivers.^1^ The procedures conducted in each patient visit were recorded using Swedish Classification of Procedures in Health Care.

The Danish National Patient Register started in 1977, recording all inpatient contacts from non-psychiatric department.^2^ From 1995, the records from psychiatric inpatient, outpatients, and emergency department were also included in the register. Data on psychiatric inpatient contacts for the period 1969-1995 were retrieved from the Psychiatric Central Research Register and merged to the patient register.^2^ Hence, for psychiatric care, we have the records of inpatient care from 1969 and outpatient care from 1995. Diagnoses were coded using the ICD 8 from 1977 to 1993, and the ICD 10 since 1994 onward. Since the Danish patient register used a modified version of the ICD 8, three-digit ICD 8 codes plus two supplementary digits, the ICD 9 were never used in the register.^2^

Multi-Generation Register:

The Swedish Multi-Generation Register records information on parents (97% mother, 95% father) of individuals who were born on and after 1932.^7^ In Denmark, the Multi-Generation Register covers parental information individuals who born on and after 1960. The identification number of parents were used to identify siblings which share parents, and first cousin who share both grandparents on either mother or father side.

Cause of death register:

The cause of death registers which record all deaths and their underlying causes using ICD codes^3,4^ were used to identify death in relation to self-harm cases in this study. The registers started in 1952 in Sweden, and in 1875 in Denmark.

The Swedish Cause of Death Register records the underlying cause of death using different versions of ICD codes including ICD-6 from 1952 to 1957, ICD-7 from 1958 to 1968, ICD-8 from 1969 to 1986, ICD-9 from 1987 to 1996, and ICD-10 from 1997 onwards. In this study, we used the ICD codes version 8, 9, 10 to identify deaths in relation to self-harm.^3^

In the Danish register, the underlying cause of death was recorded using ICD-10 codes since 1994. For the deaths registered before 1994, the underlying cause of death was given an ICD-10 code based on the medical information on the death register by specially trained coders.^4^

Prescribed drug register:

The Swedish National Prescribed Drug Register (NPR)^9^ records all prescribed drugs dispenses at pharmacies in Sweden using ATC codes since July 2005. In this study, we used NPR to collect information about antipsychotic treatment (ATC codes N05A).

## **eMethods 2. Genotype data**

For the PRS analyses, we used genotype data from two data sources including the UK-Biobank^10^, and the PREFECT study.^11^ In both datasets, there were few psychotic MDD cases of non-European ancestries, we therefore only we included individuals of European ancestries in the analyses.

The **UK-Biobank** recruited >500,000 adults within the age 37-73 who live in the United Kingdom; further details available elsewhere.^10^ Genotype data were collected for majority of the participants. Collected blood samples were genotyped using two similar arrays, Applied Biosystems UK BiLEVE Axiom Array by Affymetrix (807,411 markers) and Applied Biosystems UK Biobank Axiom Array (825,927 markers). The genotype data undergone a stringent quality control procedure, then was imputed using combined reference panels of Haplotype Reference Consortium (HRC) and UK10K merged with 1000 Genomes phase 3, which resulted in >93 millions autosomal SNPs. The genetic principal components were computed using the *fastPCA*^12^ algorithm. The top 40 PCs were first computed using 407,219 unrelated, high quality samples and 147,604 high quality markers. All UK-Biobank samples were then projected onto the computed PCs to created PC score for the whole cohort. To identify individuals of European ancestry, ancestry outliers were identified using the method published by Price et al. (2006)^13^, where any data points beyond three standard deviations from the mean were considered outliers.

The **PREFECT study** included 2,904 individuals who received electroconvulsive therapy (ECT) and were registered in the Swedish National Quality Register for ECT during the period 2013-2017.^11^ DNA was collected from peripheral blood and genotyped using the Illumina GSA-MD SNP arrays (v1). Raw genotype data was QCed using the PGC Ricopili pipeline including removing individuals with genotype missingness > 0, genotypic sex ambiguity, phenotypic mismatch, autosomal heterozygosity |F| > 0.2; and removing SNPs with call rates < 0.99, difference in missingness between cases and controls > 0.005, MAF < 0.01, deviation from Hardy–Weinberg equilibrium (*P* < 10^−6^). After QC, the data were imputed using the HRC r1.1 reference panel. To identify individuals of European ancestry, ancestry outliers (>3 standard deviations from the European reference) were removed using the principal components (PCs) generated in 1000 Genomes reference data (phase 3, version 5). Details about PREFECT data was published elsewhere.^11^

## **eMethods 3. Estimate familial aggregation and coaggregation**

We studied the familial aggregation of psychotic MDD and its co-aggregation with MDD, SCZ/SAD, BD, and the all psychotic disorders (*i.e.*, all disorders with psychotic symptoms). We also presented the familial aggregation of MDD for comparison. These analyses involve pairwise comparison, *i.e.*, one individual functioned as both proband and relative. For each type of relative (full-siblings, half-siblings, and cousins), we constructed a dataset where each pair appeared twice, with the role of proband and relative switched between the two individuals. All possible pairs in families were included.

For each type of relative, we calculated two types of odds ratios (OR) using Generalized Estimating Equation with logit link function: 1) the odds of having disorder X in a relative given X in proband compared to the odds of X in a relative given no X in proband (X could be psychotic MDD, or MDD); 2) the odds of Y in a relative given X in proband compared to the odds of Y in a relative given no X in proband (Y could be MDD, SCZ/SAD, BD or psychotic disorders; X could be psychotic MDD). Because all relative pairs were included, *i.e.*, there was non-independency between observations, robust standard errors (SE) were estimated by including family as clusters in the models. The GEE model accounts for clustering by introducing a working correlation structure that models the dependency among observations within the same families. The estimates parameters (e.g., coefficients) are consistently averaged over the clusters. The method is embedded in the function gee() of the R package drgee^14^ by specifying a cluster variable as below:

gee(trait_relative_1 ~ trait_relative_2 + sex_1 + byear_1 + sex_2 + byear_2, data=data_for_each_relative_type, link='logit', clusterid='famID')”

## **eMethods 4. Pedigree heritability and genetic correlation**

We applied structural equation models (*OpenMx* package version 2.19.8 in R^15^) to estimate heritability (*h^2^)* on liability-scale using univariate models and genetic correlations (*r_g_)* using bivariate models. The variance and covariance matrices were used to estimate three components: additive genetic (*A*), shared environment (*C*), and unique environment (*E*) which include measurement error. We did not model the interaction between genetic and environmental components within the scope of this study. Because there were too few concordant case pairs among twins and half-siblings to make robust estimation, we used data from full-siblings and cousins because they account for the majority of relative pairs in our populations. We assumed full-siblings share 50% of *A* while, cousins share 12.5%; and full-siblings share 100% of *C* while cousins do not share any *C*. To avoid underestimation of SE due to inclusion of all relative pairs in families, we applied Bootstrap resampling of families (1,000 replicates) to obtain Bootstrap SEs, which were then used for meta-analysis of Swedish and Danish estimates. The Bootstrap SEs were calculated as the square-root of the variance of the 1000 Bootstrap estimates.

For the *h^2^* estimation, we fitted both ACE and AE models using weighted least squares (WLS), and presented AE models as main results. We applied the liability threshold model to estimate *h^2^* estimates on the liability scale. For *r_g_* estimation, we fitted two types of models. We used WLS to estimate *r_g_* between traits that are non-mutually exclusive (i.e., one person can have both traits); and we compared model fit between ACE and AE using Chi-squared test. The other model is to estimate genetic correlation between traits that are mutually exclusive, psychotic MDD and non-psychotic MDD in this case. Details about model setup has been published elsewhere.^16^ The models were fit using Maximum Likelihood (ML), and we compared model fit using likelihood ratio test.

## **eMethods 5. Computing PRS**

We computed PRS of MDD, SCZ, BD, BD type-I and type-II using the latest genome-wide association study (GWAS) summary statistics.^17-19^ To avoid potential overlap in samples between discovery GWAS and the two target samples, we excluded the relevant population in the discovery set, *i.e.*, the PRS in the UKB samples were calculated from the GWAS leaving the UKB out, and similarly, the PRS in the Swedish cohort PREFECT were calculated using summary statistics for each disorder without any Swedish samples (Details in *eTable 3*).

The GWAS summary statistics were first QCed to remove duplicate SNPs, rare variants (MAF <0.01) and low-quality imputed SNPs if available (INFO score <0.9).

Second, we used the model SBayesR^20^ of the GCTB software (https://cnsgenomics.com/software/gctb/#Overview) to rescale the SNP effects, accounting for LD. The SNPs in the major histocompatibility complex regions (chr6:28-34 Mb) were also removed. The parameters used are:

--bayes R

--pi 0.95, 0.2, 0.2, 0.1

--gamma 0, 0.1, 0.1, 1

--exclude-mhc

--chain-length 10000

--burn-in 4000

Last, the PRS was computed using the --score command in the software PLINK 2.0 (www.cog-genomics.org/plink/2.0/). PRS is the sum of all the weighted effect of SNPs from the rescaled summary statistics.^21^ Each PRS were standardized, *i.e.,* linearly transformed the variant's dosage vector to have mean 0, variance 1, before being used in analyses.

## **eMethods 6. Meta-analyses**

We used fixed effect model^22^ to conduct meta-analyses assuming estimates from different data were actually from a single distribution, and the differences in the estimates were due to sampling error.

For each sample *i* in the study, we have an estimate $E_{i}$ and a variance $v_{i}$ which is the squared standard error (SE) of the estimate $E_{i}$

We used the inverse variance method to assign weight to each dataset:

$$w_{i}= \frac{1}{v_{i}}$$

where

$w_{i}$ = weight assigned to the dataset *i*

$v_{i}$ = variance of the estimate *i*

The combined estimates $E_{combined}$ from samples *i* was calculated as

$$E_{combined}=\frac{\sum_{i}^{k} w_{i} E_{i}}{\sum_{i}^{k} w_{i}}$$

where

*k* = number of included datasets

$w_{i}$ = weight assigned to the dataset *i*

$E_{i}$ = estimate from the dataset *i*

The variance ($v_{combined})$ and standard error (${SE}_{combined}$) of the combined estimates were defined as:

$${SE}_{combined}=\sqrt{v_{combined}}= \sqrt{\frac{1}{\sum_{i}^{k} w_{i}}}$$

where

*k* = number of included datasets

$w_{i}$ = weight assigned to the dataset *i*

The 95% confidence interval (CI) for the combined estimates was computed as:

$$Lower limit= E_{combined}-1.96*{SE}_{combined}$$

$$Upper limit= E_{combined}+1.96*{SE}_{combined}$$

# **Supplementary tables**

## **eTable 1:** Phenotype definitions in each dataset

| **Phenotype** | **Register data** | **PREFECT** | **UKB** | |  |
| --- | --- | --- | --- | --- | --- |
| **MDD** | Any MDD diagnosis using ICD 8, 9, 10 | Any MDD diagnosis - F32/F33 | Any MDD diagnosis (F32/F33) (data fields 41202 & 41204 recorded primary and secondary diagnoses from hospital records) | |  |
| **Non-MDD** | People without any MDD diagnosis* | N/A | N/A | |  |
| **Psychotic MDD** | Any psychotic MDD diagnosis F32.3, F33.3 | Any psychotic MDD diagnosis F32.3, F33.3 | Any psychotic MDD diagnosis F32.3, F33.3 | |  |
| **Non-psychotic MDD** | Other MDD diagnoses that are not psychotic MDD using ICD 10 | Other MDD cases | Other MDD cases | |  |
| **For sensitivity analyses** | | | | | |
| **Restrictive psychotic MDD** | Any psychotic MDD diagnosis F32.3, F33.3  Excluding lifetime diagnosis of bipolar disorder, schizophrenia, schizoaffective disorder using ICD 8, 9, 10 | Any psychotic MDD diagnosis F32.3, F33.3  Excluding lifetime diagnosis of bipolar disorder, schizophrenia, schizoaffective disorder using ICD 8, 9, 10 | Any psychotic MDD diagnosis F32.3, F33.3  Excluding lifetime diagnosis of bipolar disorder, schizophrenia, schizoaffective disorder using ICD 10 |  |  |
| **Restrictive non-psychotic MDD** | Other MDD diagnoses that are not psychotic MDD using ICD 10  Excluding lifetime diagnosis of bipolar disorder using ICD 8, 9, 10 | Other MDD cases  Excluding lifetime diagnosis of bipolar disorder using ICD 8, 9, 10 | Other MDD cases  Excluding lifetime diagnosis of bipolar disorder using ICD 10 |  |  |

*In all data sources, we used specialist-diagnosis to ascertain psychiatric disorders. Detailed ICD codes in eTable 2.* ***MDD*** *cases were identified using ICD codes version 8, 9, 10 for Swedish register data; version 8 and 10 for the Danish register data (version 9 was not adopted by Denmark), and version 10 for PREFECT and UK-Biobank. Among MDD cases, we identified* ***psychotic MDD*** *as having at least one psychotic MDD diagnosis (ICD-10 codes F32.3 or F33.3).*

**Individuals without MDD were identified in the register data for the analyses of familial risk, pedigree heritability and genetic correlations. Because psychotic MDD could only be identified using ICD-10 codes, when comparing non-MDD with the psychotic MDD, individuals with only ICD 8-9 MDD diagnoses were excluded from both case and non-case groups in these analyses.*

*For sensitivity analyses, we derived a restrictive definition aiming to limit the impact of psychotic MDD within the context of bipolar disorder, schizophrenia, or schizoaffective disorder. We applied the exclusion criteria reported in a previous study^23^: excluding bipolar disorder from all MDD cases, and further excluding SCZ and SAD from psychotic MDD cases.*

## **eTable 2**: ICD codes

| **Disorders** | **ICD codes*** | **ICD 9*** | **ICD 8*** |  |
| --- | --- | --- | --- | --- |
| Major depressive disorder (MDD) | F32 MDD single  F33 MDD recurrent | 296B Episodic mood disorders, MDD recurrent  311 MDD not classified elsewhere | 300.4 Depressive neuroses |  |
| Psychotic MDD | F32.3 severe MDD with psychotic symptoms, single  F33.3 severe MDD with psychotic symptoms, recurrent | N/A | N/A |  |
| Bipolar, manic disorder | F30 Mania  F31 Bipolar affective disorder | 296A Bipolar I disorder, single manic episode  296C Bipolar affective psychosis, manic phase  296D Manic disorder, recurrent episode  296E Bipolar I disorder, most recent episode (or current) manic  296F Bipolar I disorder, most recent episode (or current) depressed  296G Bipolar I disorder, most recent episode (or current) mixed  296H Bipolar I disorder, most recent episode (or current) unspecified  296W Other and unspecified bipolar disorders  296X Other and unspecified episodic mood disorder | 296.1 Manic-depression psychosis, manic type  296.2 Manic depressive psychosis, depressed type  296.3 Manic-depressive psychosis, circular type  296.8 Mental disorders, affective psychosis, other  296.9 Mental disorders, affective psychosis, unspecified |  |
| Schizophrenia | F20.0 Paranoid schizophrenia  F20.1 Hebephrenic schizophrenia  F20.2 Catatonic schizophrenia  F20.3 Undifferentiated schizophrenia  F20.4 Post-schizophrenic depression  F20.5 Residual schizophrenia  F20.6 Simple schizophrenia  F20.8 Other schizophrenia  F20.9 Schizophrenia, unspecified | 295A Schizophrenic disorders, simple type  295B Disorganized type  295C Catatonic type  295D Paranoid type  295E Schizophreniform disorder  295G Residual type  295W Other specified types of schizophrenia  295X Unspecified schizophrenia | 295.0 Schizophrenia, simple type  295.1 Hebephrenic type  295.2 Catatonic type  295.3 Paranoid type  295.4 Acute schizophrenia episode  295.6 Residual schizophrenia  295.8 Other  295.9 Unspecified type |  |
| Schizoaffective disorder | F25.0 Schizoaffective disorder, manic type  F25.1 Schizoaffective disorder, depressive type  F25.2 Schizoaffective disorder, mixed type  F25.8 Other schizoaffective disorders  F25.9 Schizoaffective disorder, unspecified | 295H Schizoaffective disorder | 295.7 Mental disorders, Schizophrenia, Schizoaffective type |  |
| Other psychotic disorders | F21 Schizotypal disorder  F22 Persistent delusional disorders  F23 Acute and transient psychotic disorders  F24 Induced delusional disorder  F28 Other nonorganic psychotic disorders  F29 Unspecified nonorganic psychosis | 297 Delusional disorders  297B Delusional disorders, Paranoid state, simple 297C Delusional disorders, Paraphrenia 297D Delusional disorders, Induced psychotic disorder 297W Delusional disorders, Other specified paranoid states 297X Delusional disorders, Unspecified paranoid state  298A Depressive type psychosis  298B Excitative type psychosis  298C Acute paranoid reaction  298E Psychogenic paranoid psychosis  298W Other nonorganic psychoses, Other and unspecified reactive psychosis  298X Other nonorganic psychoses, Unspecified psychosis | 297.0 Paranoid states, Paranoia 297.1 Paranoid states, Involutional paraphrenia 297.9 Paranoid states, Other  298.0 Reactive depressive psychosis 298.3 Other psychoses, Acute paranoid reaction 298.9 Other psychoses, Reactive psychosis, unspecified |  |
|  |  |  |  |  |
|  |  |  |  |  |
|  |  |  |  |  |
|  |  |  |  |  |
|  |  |  |  |  |
|  |  |  |  |  |
|  |  |  |  |  |
|  |  |  |  |  |
|  |  |  |  |  |
|  |  |  |  |  |
|  |  |  |  |  |
|  |  |  |  |  |
|  |  |  |  |  |
|  |  |  |  |  |
|  |  |  |  |  |
| Anxiety | F40.0 Agoraphobia  F40.1 Social phobias  F40.2 Specific (isolated) phobias  F40.8 Other phobic anxiety disorders  F40.9 Phobic anxiety disorder, unspecified  F41.0 Panic disorder [episodic paroxysmal anxiety]  F41.1 Generalized anxiety disorder  F41.2 Mixed anxiety and depressive disorder  F41.3 Other mixed anxiety disorders  F41.8 Other specified anxiety disorders  F41.9 Anxiety disorder, unspecified | 300.00 Anxiety state, unspecified  300.01 Panic disorder without agoraphobia  300.02 Generalized anxiety disorder  300.09 Other anxiety states  300.20 Phobia, unspecified  300.21 Agoraphobia with panic disorder  300.22 Agoraphobia without mention of panic attacks  300.23 Social phobia  300.29 Other isolated or specific phobias | 300.0 Anxiety neurosis  300.2 Phobic neurosis |  |
| Eating disorder | F50.0 Anorexia nervosa  F50.1 Atypical anorexia nervosa  F50.2 Bulimia nervosa  F50.3 Atypical bulimia nervosa  F50.4 Overeating associated with other psychological disturbances  F50.5 Vomiting associated with other psychological disturbances  F50.8 Other eating disorders  F50.9 Eating disorder, unspecified | 307.1 Anorexia nervosa  307.5 Other and unspecified disorders of eating | 784 Anorexia  306.5 Feeding disturbance |  |
| Substance use disorders (not including tobacco) | F10 Alcohol related disorders  F11 Opioid related disorders  F12 Cannabis related disorders  F13 Sedative, hypnotic, or anxiolytic related disorders  F14 Cocaine related disorders  F15 Other stimulant related disorders  F16 Hallucinogen related disorders  F18 Inhalant related disorders  F19 Other psychoactive substance related disorders | 291.0 Alcohol withdrawal delirium  291.1 Alcohol-induced persisting amnestic disorder  291.2 Alcohol-induced persisting dementia  291.3 Alcohol-induced psychotic disorder with hallucinations  291.4 Idiosyncratic alcohol intoxication  291.5 Alcohol-induced psychotic disorder with delusions  291.8 Other specified alcohol-induced mental disorders  291.9 Unspecified alcohol-induced mental disorders  292.0 Drug withdrawal  292.1 Drug-induced psychotic disorders  292.2 Pathological drug intoxication  292.8 Other specified drug-induced mental disorders  292.9 Unspecified drug-induced mental disorder  303.0 Acute alcoholic intoxication  303.9 Other and unspecified alcohol dependence  304.0 Opioid type dependence  304.1 Sedative, hypnotic or anxiolytic dependence  304.2 Cocaine dependence  304.3 Cannabis dependence  304.4 Amphetamine and other psychostimulant dependence  304.5 Hallucinogen dependence  304.6 Other specified drug dependence  304.7 Combinations of opioid type drug with any other  304.8 Combinations of drug dependence excluding opioid type drug  304.9 Unspecified drug dependence  305.0 Alcohol abuse  305.2 Cannabis abuse  305.3 Hallucinogen abuse  305.4 Sedative, hypnotic or anxiolytic abuse  305.5 Opioid abuse  305.6 Cocaine abuse  305.7 Amphetamine or related acting sympathomimetic abuse  305.9 Other, mixed, or unspecified drug abuse | 291.0 Delirium tremens  291.1 Korsakov's psychosis  291.2 Other alcoholic hallucinosis  291.3 Alcoholic paranoia  291.9 Other and unspecified  303.0 Episodic excessive drinking  303.1 Habitual excessive drinking  303.2 Alcoholic addiction  303.9 Other and unspecified alcoholism  304.0 Opium, opium alkaloids, and their derivatives  304.1 Synthetic analgesics with morphine-like effects  304.2 Barbiturates  304.3 Other hypnotics and sedatives or tranquilizers  304.4 Cocaine  304.5 Cannabis sativa  304.6 Other psycho-stimulants  304.7 Hallucinogenic  304.8 Drug dependence, other  304.9 Drug dependence, unspecified |  |
| Attention deficit hyperactivity disorder (ADHD) | F90.0 Disturbance of activity and attention  F90.1 Hyperkinetic conduct disorder  F90.8 Other hyperkinetic disorders | 314.01 Attention deficit disorder with hyperactivity  314.1 Hyperkinesis with developmental delay  314.2 Hyperkinetic conduct disorder  314.8 Other specified manifestations of hyperkinetic syndrome  314.9 Unspecified hyperkinetic syndrome | N/A |  |
| Autism spectrum disorder (ASD) | F84.0 Childhood autism  F84.1 Atypical autism  F84.5 Asperger's syndrome | 299.0 Autistic disorder | N/A |  |
| Obsessive-compulsive disorder (OCD) | F42.0 Predominantly obsessional thoughts or ruminations  F42.1 Predominantly compulsive acts [obsessional rituals]  F42.2 Mixed obsessional thoughts and acts  F42.8 Other obsessive-compulsive disorders  F42.9 Obsessive-compulsive disorder, unspecified | 300.3 Obsessive-compulsive disorders | 300.3 Obsessive compulsive neurosis |  |
| Post-traumatic stress disorder (PTSD) | F43.0 Acute stress reaction  F43.1 Posttraumatic stress disorder  F43.2 Adjustment disorders  F43.8 Other reactions to severe stress  F43.9 Reaction to severe stress, unspecified | 308.0 Predominant disturbance of emotions  308.1 Predominant disturbance of consciousness  308.2 Predominant psychomotor disturbance  308.3 Other acute reactions to stress  308.4 Mixed disorders as reaction to stress  308.9 Unspecified acute reaction to stress  309.0 Adjustment disorder with depressed mood  309.1 Prolonged depressive reaction  309.2 Adjustment reaction with predominant disturbance of other emotions  309.3 Adjustment disorder with disturbance of conduct  309.4 Adjustment disorder with mixed disturbance of emotions and conduct  309.8 Other specified adjustment reactions  309.9 Unspecified adjustment reaction | 307 Transient situation disturbances |  |
| Intentional self-harm (from patient register and cause of death register) | X60 Intentional self-poisoning by, exposure to nonopioid analgesics, antipyretics, and antirheumatics  X61 Intentional self-poisoning by and exposure to antiepileptic, sedative-hypnotic, anti-parkinsonism, and psychotropic drugs, not elsewhere classified  X62 Intentional self-poisoning by and exposure to narcotics and psychodysleptics, not elsewhere classified  X63 Intentional self-poisoning by, exposure to other drugs acting on the autonomic nervous system  X64 Intentional self-poisoning by, exposure to other/unspecified drugs, medicaments, biological substances  X65 Intentional self-poisoning by, exposure to alcohol  X66 Intentional self-poisoning by, exposure to organic solvents, halogenated hydrocarbons and their vapors  X67 Intentional self-poisoning by, exposure to other gases and vapors  X68 Intentional self-poisoning by, exposure to pesticides  X69 Intentional self-poisoning by, exposure to other and unspecified chemicals and noxious substances  X70 Intentional self-harm by hanging, strangulation, and suffocation  X71 Intentional self-harm by drowning and submersion  X72 Intentional self-harm by handgun discharge  X73 Intentional self-harm by rifle, shotgun, larger firearm discharge  X74 Intentional self-harm by other and unspecified firearm discharge  X75 Intentional self-harm by explosive material  X76 Intentional self-harm by smoke, fire, and flames  X77 Intentional self-harm (suicide) by steam, hot vapors, and hot objects  X78 Intentional self-harm by sharp object  X79 Intentional self-harm by blunt object  X80 Intentional self-harm by jumping from a high place  X81 Intentional self-harm by jumping or lying before moving object  X82 Intentional self-harm by crashing of motor vehicle  X83 Intentional self-harm by other specified means  X84 Intentional self-harm by unspecified means | E950 Suicide and self-inflicted poisoning by solid or liquid substances  E951 Suicide and self-inflicted poisoning by gases in domestic use  E952 Suicide and self-inflicted poisoning by other gases and vapors  E953 Suicide and self-inflicted injury by hanging strangulation and suffocation  E954 Suicide and self-inflicted injury by submersion [drowning]  E955 Suicide and self-inflicted injury by firearms air guns and explosives  E956 Suicide and self-inflicted injury by cutting and piercing instrument  E957 Suicide and self-inflicted injuries by jumping from high place  E958 Suicide and self-inflicted injury by other and unspecified means  E959 Late effects of self-inflicted injury | E950 Suicide and self-inflicted poisoning by solid or liquid substances  E951 Suicide and self-inflicted poisoning by gases in domestic use  E952 Suicide and self-inflicted poisoning by other gases  E953 Suicide and self-inflicted injury by hanging, strangulation, and suffocation  E954 Suicide and self-inflicted injury by submersion (drowning)  E955 Suicide and self-inflicted injury by firearms and explosives  E956 Suicide and self-inflicted injury by cutting and piercing instruments  E957 Suicide and self-inflicted injury by jumping from high place  E958 Suicide and self-inflicted injury by other and unspecified means  E959 Late effect of self-inflicted injury |  |
| **When the last character in the code was not specified, all sub-codes starting with the specified characters were included* | | | | |

## **eTable 3**: Summary statistics used for PRS analyses

| **PRS for** | **GWAS summary statistics** | **PMID** | **Note** |
| --- | --- | --- | --- |
| PREFECT | MDD 2018, no UK-Biobank, no 23&me | 29700475^17^ | Original study did not include PREFECT samples |
|  | SCZ 2022 excl. Sweden | 35396580^18^ | Requested data |
|  | BD 2021 excl. Sweden | 34002096^19^ | Requested data |
|  | BD type-I 2021 excl. Sweden | 34002096^19^ | Requested data |
|  | BD type-II 2021 excl. Sweden | 34002096^19^ | Requested data |
| UKB | MDD 2018, no UK-Biobank, no 23me | 29700475^17^ | Original GWAS did not include UK-Biobank |
|  | SCZ 2022 | 35396580^18^ | Original GWAS did not include UK-Biobank |
|  | BD 2021 excl. UK-Biobank | 34002096^19^ | Requested data |
|  | BD type-I 2021 excl. UK-Biobank | 34002096^19^ | Original GWAS did not include UK-Biobank |
|  | BD type-II 2021 excl. UK-Biobank | 34002096^19^ | Original GWAS did not include UK-Biobank |

## **eTable 4**: Proportion of psychotic MDD and conversion rate to other psychotic disorders

| 1. **Compare proportion of psychotic MDD between Sweden and Denmark** | | | |
| --- | --- | --- | --- |
|  | **Swedish register** | **Danish register** | **Compare Sweden and Denmark** |
| Psychotic MDD | 5,597 | 4,575 |  |
| MDD (ICD 10) | 168,186 | 87,940 | χ^2^=531.57, df = 1, p-value < 2.2*10^-16^  95% CI for difference -0.020; -0.017 |
| % psychotic MDD among MDD | 3.33% | 5.20% |  |
| Total included population | 3,171,281 | 1,947,391 | χ^2^=207.46, df = 1, p-value < 2.2*10^-16^  95% CI for difference -0.000667; -0.000502 |
| % psychotic MDD in population | 0.18% | 0.23% |  |

1. **Conversion rate of between psychotic MDD and SCZ/SAD, BD in register data**

|  | **Swedish register** | | **Danish register** | | **Combined 2 countries** | |  |
| --- | --- | --- | --- | --- | --- | --- | --- |
|  | **Psychotic MDD**  (N=5597) | **Non-psychotic MDD**  (N=168145) | **Psychotic MDD**  (N=4575) | **Non-psychotic MDD**  (N=83365) | **Psychotic MDD**  (N=10172) | **Non-psychotic MDD**  (N=251510) |  |
| **Conversion from/to schizophrenia/schizoaffective disorder** | | | | | | | |
| Had SCZ/SAD **before** first MDD | 176 (3.14%) | 1254  (0.75%) | 185  (4.04%) | 1353  (1.62%) | 361  (3.56%) | 2607  (1.04%) |  |
| Had SCZ/SAD **after** first MDD | 743 (13.27%) | 3217  (1.91%) | 873 (19.08%) | 3101  (3.72%) | 1616  (15.92%) | 6318  (2.51%) |  |
| **Conversion from/to BD** | | | | | | | |
| Had BD **before** first MDD | 219 (3.91%) | 3416  (2.03%) | 99  (2.16%) | 939  (1.13%) | 318  (3.13%) | 4355  (1.73%) |  |
| Had BD **after** first MDD | 1203 (21.49%) | 14823  (8.82%) | 563 (12.28%) | 3832  (4.60%) | 1766  (17.40%) | 18655  (7.42%) |  |
| **Conversion from/to other psychotic disorders (F2 excl. F20/F25)** | | | | | | | |
| Had other psychotic disorders **before** first MDD | 723 (12.92%) | 3751  (2.23%) | 461 (10.07%) | 2493  (2.99%) | 1184  (11.66%) | 6244  (2.48%) |  |
| Had other psychotic disorders **after** first MDD | 1497 (26.75%) | 5792  (3.44%) | 726 (15.87%) | 3280  (3.93%) | 2223  (21.90%) | 9072  (3.61%) |  |
| **No conversion** | | | | | | | |
| Did **not** have SCZ/SAD, BD or other psychotic disorders **before** first MDD | 4676 (83.54%) | 160981 (95.74%) | 3968 (86.73%) | 79583  (95.46%) | 8644  (85.15%) | 240564  (95.65%) |  |
| Did **not** have SCZ/SAD, BD or other psychotic disorders **after** first MDD | 3046 (54.42%) | 147680 (87.83%) | 2936 (64.17%) | 74885  (89.83 %) | 5982  (58.93%) | 222565  (88.49%) |  |
| Did **not** have **life-time** SCZ/SAD, BD or other psychotic disorders | 2800  (50.03%) | 144715 (86.07%) | 2374 (51.9%) | 70974  (85.13%) | 5174  (50.97%) | 215689  (85.76%) |  |

*^a^Diagnosis in primary care were not included in register data. In many cases, MDD diagnoses could have been given in primary care. MDD: Major depressive disorder | SCZ/SAD: Schizophrenia/schizoaffective disorder | BD: bipolar disorder*

## **eTable 5**: Number of concordant/discordant pairs of MDD phenotypes in register data

|  | | | **Sibling/cousin 2** | | | | | | | | |
| --- | --- | --- | --- | --- | --- | --- | --- | --- | --- | --- | --- |
|  |  |  | **Full-sibling** | | | **Half-sibling** | | | **Cousin** | | |
| **Sweden** | **MDD ICD 10*** | **Sibling/cousin 1** | **No** | **Yes** | **N/A** | **No** | **Yes** | **N/A** | **No** | **Yes** | **N/A** |
|  | No |  | 1768345 | 90073 | 3180 | 322902 | 2816 | 942 | 5131236 | 279468 | 7133 |
|  | Yes |  | 89737 | 10666 | 346 | 27930 | 3513 | 124 | 278738 | 19614 | 462 |
|  | NA |  | 3093 | 322 | 58 | 924 | 120 | 9 | 7104 | 471 | 32 |
|  | **Psychotic MDD** |  | **No** | **Yes** |  | **No** | **Yes** |  | **No** | **Yes** |  |
|  | No |  | 1958640 | 3523 |  | 382838 | 887 |  | 5705188 | 9473 |  |
|  | Yes |  | 3640 | 17 |  | 897 | 8 |  | 9576 | 21 |  |
|  | **MDD ICD 8910** |  | **No** | **Yes** |  | **No** | **Yes** |  | **No** | **Yes** |  |
|  | No |  | 1768345 | 93253 |  | 322902 | 29108 |  | 5131236 | 286601 |  |
|  | Yes |  | 92830 | 11392 |  | 28854 | 3766 |  | 285842 | 20579 |  |
| **Denmark** | **MDD ICD 10*** | **Sibling/cousin 1** | **No** | **Yes** | **N/A** | **No** | **Yes** | **N/A** | **No** | **Yes** | **N/A** |
|  | No |  | 1115579 | 48340 | 349 | 221107 | 15864 | 100 | 1100218 | 51041 | 19 |
|  | Yes |  | 48622 | 4671 | 33 | 16104 | 1623 | 4 | 50772 | 3144 | 2 |
|  | NA |  | 404 | 30 | 2 | 97 | 9 | 0 | 19 | 1 | 0 |
|  | **Psychotic MDD** |  | **No** | **Yes** |  | **No** | **Yes** |  | **No** | **Yes** |  |
|  | No |  | 1212138 | 2907 |  | 253177 | 878 |  | 1200508 | 2364 |  |
|  | Yes |  | 2951 | 34 |  | 846 | 7 |  | 2338 | 6 |  |
|  | **MDD ICD 8910** |  | **No** | **Yes** |  | **No** | **Yes** |  | **No** | **Yes** |  |
|  | No |  | 1115579 | 48689 |  | 221107 | 15964 |  | 1100218 | 51060 |  |
|  | Yes |  | 49026 | 4736 |  | 16201 | 1636 |  | 50791 | 3147 |  |

*N/A (missing value) only exists in MDD defined by ICD 10, indicating those who had diagnosis in ICD 8/9 but not in ICD 10

## **eTable 6**: Familial aggregation separately for Sweden and Denmark

|  | **Sweden** | | | | | | **Denmark** | | | | | | | | | **Combined estimates** | | | | | | | |
| --- | --- | --- | --- | --- | --- | --- | --- | --- | --- | --- | --- | --- | --- | --- | --- | --- | --- | --- | --- | --- | --- | --- | --- |
|  | **Coefficient** | **SE** | **p-value** | **OR** | **95% CI OR** | | **Coefficient** | **SE** | **p-value** | | **OR** | | **95% CI OR** | | | **Coefficient** | **SE** | | **OR** | | **95% CI OR** | | |
|  | **Full-sibling** | | | | | | **Full-sibling** | | | | | | | | | **Full-sibling** | | | | | | | |
| MDD ICD 10 | 0.85 | 0.01 | <2.2*10^-308^ | 2.33 | 2.28; | 2.38 | 0.79 | 0.02 | | <2.2*10^-308^ | | 2.21 | | 2.14; | 2.28 | 0.83 | | 0.01 | | 2.29 | | 2.25; | 2.33 |
| Psychotic MDD | 0.92 | 0.24 | 1.40*10^-04^ | 2.52 | 1.57; | 4.05 | 1.57 | 0.16 | | 7.38*10^-24^ | | 4.78 | | 3.53; | 6.49 | 1.38 | | 0.13 | | 3.97 | | 3.07; | 5.13 |
|  |  |  |  |  |  |  |  |  | |  | |  | |  |  | Compare aggregation MDD 10 – Psychotic MDD^a^: p-value=**2.72*10^-05§^** | | | | | | | |
| MDD ICD 8, 9, 10 | 0.85 | 0.01 | <2.2*10^-308^ | 2.33 | 2.28; | 2.38 | 0.79 | 0.02 | | <2.2*10^-308^ | | 2.20 | | 2.13; | 2.27 | 0.83 | | 0.01 | | 2.29 | | 2.25; | 2.33 |
|  | **Half-sibling** | | | | | | **Half-sibling** | | | | | | | | | **Half-sibling** | | | | | | | |
| MDD ICD 10 | 0.37 | 0.02 | 3.95*10^-74^ | 1.44 | 1.39; | 1.50 | 0.33 | 0.03 | | 8.85*10^-32^ | | 1.39 | | 1.32; | 1.47 | 0.35 | | 0.02 | | 1.42 | | 1.38; | 1.47 |
| Psychotic MDD | 1.35 | 0.39 | 5.94*10^-04^ | 3.85 | 1.78; | 8.31 | 0.79 | 0.38 | | 3.77*10^-02^ | | 2.19 | | 1.05; | 4.60 | 1.06 | | 0.27 | | 2.88 | | 1.69; | 4.90 |
|  |  |  |  |  |  |  |  |  | |  | |  | |  |  | Compare aggregation MDD 10 – Psychotic MDD^a^: p-value=**1.15*10^-02§^** | | | | | | | |
| MDD ICD 8, 9, 10 | 0.38 | 0.02 | 4.45*10^-83^ | 1.46 | 1.40; | 1.51 | 0.33 | 0.03 | | 2.01*10^-31^ | | 1.38 | | 1.31; | 1.46 | 0.36 | | 0.02 | | 1.43 | | 1.39; | 1.48 |
|  | **Cousin** | | | | | | **Cousin** | | | | | | | | | **Cousin** | | | | | | | |
| MDD ICD 10 | 0.25 | 0.01 | 6.31*10^-185^ | 1.29 | 1.26; | 1.31 | 0.28 | 0.02 | | 1.73*10^-42^ | | 1.32 | | 1.27; | 1.38 | 0.26 | | 0.01 | | 1.29 | | 1.27; | 1.31 |
| Psychotic MDD | 0.27 | 0.22 | 2.08*10^-01^ | 1.31 | 0.86; | 2.01 | 0.33 | 0.38 | | 3.74*10^-01^ | | 1.40 | | 0.67; | 2.91 | 0.29 | | 0.19 | | 1.33 | | 0.92; | 1.92 |
|  |  |  |  |  |  |  |  |  | |  | |  | |  |  | Compare aggregation MDD 10 – Psychotic MDD^a^: p-value=8.65*10^-01^ | | | | | | | |
| MDD ICD 8, 9, 10 | 0.25 | 0.01 | 3.44*10^-193^ | 1.29 | 1.27; | 1.31 | 0.28 | 0.02 | | 1.67*10^-42^ | | 1.32 | | 1.27; | 1.38 | 0.26 | | 0.01 | | 1.29 | | 1.27; | 1.31 |

^a^Post-hoc calculation to test for differences in the 2 estimates under the assumption of independency: $\Delta e={estimate}_{1}-{estimate}_{2}$; $SE_{\Delta}=\sqrt{SE_{1}^{2}+SE_{2}^{2}}$ ; z-value = $z_{\Delta}=\frac{\Delta e}{SE_{\Delta}}$; p-value = $2\cdot\Phi\left( -abs\left( z_{\Delta} \right) \right)$

**^§^**Statistically significant at Bonferroni corrected p-value 0.05/3=0.017

## **eTable 7**: Familial coaggregation separately for Sweden and Denmark

|  | **Sweden** | | | | | | **Denmark** | | | | | | **Combined estimates** | | | | |
| --- | --- | --- | --- | --- | --- | --- | --- | --- | --- | --- | --- | --- | --- | --- | --- | --- | --- |
|  | **Coefficient** | **SE** | **p-value** | **OR** | **95% CI OR** | | **Coefficient** | **SE** | **p-value** | **OR** | **95% CI OR** | | **Coefficient** | **SE** | **OR** | **95% CI OR** | |
| **Coaggregation with MDD** | | | | | | | | | | | | | | | | | |
| **Full-sibling** | 0.81 | 0.04 | 1.87*10^-98^ | 2.25 | 2.08; | 2.42 | 0.89 | 0.05 | 3.02*10^-81^ | 2.43 | 2.22; | 2.66 | 0.84 | 0.03 | 2.32 | 2.19; | 2.46 |
| **Half-sibling** | 0.40 | 0.08 | 2.84*10^-07^ | 1.49 | 1.28; | 1.73 | 0.34 | 0.08 | 3.52*10^-05^ | 1.41 | 1.20; | 1.66 | 0.37 | 0.06 | 1.45 | 1.30; | 1.62 |
| **Cousin** | 0.16 | 0.03 | 1.26*10^-06^ | 1.17 | 1.10; | 1.25 | 0.38 | 0.06 | 5.53*10^-10^ | 1.46 | 1.30; | 1.65 | 0.21 | 0.03 | 1.23 | 1.16; | 1.30 |
| **Coaggregation with schizophrenia/schizoaffective disorder** | | | | | | | | | | | | | | | | | |
| **Full-sibling** | 1.15 | 0.10 | 3.37*10^-31^ | 3.15 | 2.60; | 3.83 | 1.07 | 0.08 | 1.33*10^-39^ | 2.92 | 2.49; | 3.42 | 1.10 | 0.06 | 3.01 | 2.66; | 3.41 |
| **Half-sibling** | 0.79 | 0.21 | 1.16*10^-04^ | 2.20 | 1.47; | 3.29 | 0.47 | 0.14 | 6.60*10^-04^ | 1.59 | 1.22; | 2.08 | 0.57 | 0.11 | 1.76 | 1.41; | 2.20 |
| **Cousin** | 0.29 | 0.10 | 3.92*10^-03^ | 1.34 | 1.10; | 1.63 | 0.41 | 0.12 | 6.27*10^-04^ | 1.51 | 1.19; | 1.92 | 0.34 | 0.08 | 1.41 | 1.21; | 1.64 |
| **Coaggregation with bipolar disorder** | | | | | | | | | | | | | | | | | |
| **Full-sibling** | 0.94 | 0.08 | 4.79*10^-32^ | 2.56 | 2.19; | 2.99 | 0.84 | 0.11 | 1.62*10^-14^ | 2.32 | 1.87; | 2.87 | 0.90 | 0.06 | 2.47 | 2.18; | 2.80 |
| **Half-sibling** | 0.54 | 0.15 | 4.30*10^-04^ | 1.71 | 1.27; | 2.31 | 0.40 | 0.21 | 5.57*10^-02^ | 1.50 | 0.99; | 2.27 | 0.49 | 0.12 | 1.64 | 1.28; | 2.09 |
| **Cousin** | 0.17 | 0.07 | 2.05*10^-02^ | 1.18 | 1.03; | 1.36 | 0.13 | 0.19 | 4.82*10^-01^ | 1.14 | 0.79; | 1.64 | 0.16 | 0.07 | 1.18 | 1.03; | 1.34 |
| **Coaggregation with psychotic disorder** | | | | | | | | | | | | | | | | | |
| **Full-sibling** | 1.06 | 0.07 | 1.90*10^-50^ | 2.88 | 2.50; | 3.30 | 1.04 | 0.06 | 5.38*10^-63^ | 2.82 | 2.50; | 3.18 | 1.04 | 0.05 | 2.84 | 2.60; | 3.11 |
| **Half-sibling** | 0.80 | 0.15 | 1.73*10^-07^ | 2.22 | 1.65; | 2.99 | 0.40 | 0.11 | 4.91*10^-04^ | 1.49 | 1.19; | 1.86 | 0.54 | 0.09 | 1.72 | 1.44; | 2.06 |
| **Cousin** | 0.28 | 0.07 | 6.65*10^-05^ | 1.32 | 1.15; | 1.51 | 0.45 | 0.09 | 1.65*10^-06^ | 1.57 | 1.30; | 1.88 | 0.34 | 0.06 | 1.40 | 1.26; | 1.57 |

## **eTable 8**: Intraclass correlation for MDD phenotypes in register data

|  | | **Sweden** | **Denmark** | **Combined** |
| --- | --- | --- | --- | --- |
| **Full-sibling** | | | | |
|  |  | N = 1965820 pairs | N = 609015 pairs |  |
| All MDD ICD 10 | Correlation | 0.198 | 0.175 | 0.190 |
|  | SE | 0.003 | 0.004 | 0.002 |
|  | 95% CI | 0.192; 0.203 | 0.168; 0.183 | 0.185; 0.194 |
| Psychotic MDD | Correlation | 0.101 | 0.190 | 0.156 |
|  | SE | 0.028 | 0.022 | 0.017 |
|  | 95% CI | 0.046; 0.156 | 0.147; 0.232 | 0.123; 0.190 |
| All MDD ICD 8, 9, 10 | Correlation | 0.199 | 0.175 | 0.191 |
|  | SE | 0.003 | 0.004 | 0.002 |
|  | 95% CI | 0.194; 0.204 | 0.168; 0.183 | 0.186; 0.195 |
| **Cousin** | | | | |
|  |  | N = 5724258 pairs | N = 602608 pairs |  |
| All MDD ICD 10 | Correlation | 0.057 | 0.060 | 0.057 |
|  | SE | 0.002 | 0.004 | 0.002 |
|  | 95% CI | 0.053; 0.060 | 0.052; 0.068 | 0.054; 0.060 |
| Psychotic MDD | Correlation | 0.027 | 0.037 | 0.030 |
|  | SE | 0.022 | 0.040 | 0.019 |
|  | 95% CI | -0.016; 0.070 | -0.041; 0.115 | -0.008; 0.067 |
| All MDD ICD 8, 9, 10 | Correlation | 0.057 | 0.061 | 0.057 |
|  | SE | 0.002 | 0.004 | 0.002 |
|  | 95% CI | 0.053; 0.060 | 0.052; 0.068 | 0.054; 0.060 |

## **eTable 9:** Heritability estimates separately for Sweden and Denmark

|  | **Sweden** | | | | | **Denmark** | | | | | **Combined estimates** | | | | **Compare estimates from Sweden and Denmark^c^**  **(p-value)** |
| --- | --- | --- | --- | --- | --- | --- | --- | --- | --- | --- | --- | --- | --- | --- | --- |
| **Phenotype** | ***h^2^*** | **SE^a^** | **95% CI^a^** | | **Weight_Swe_^b^** | ***h^2^*** | **SE** | **95% CI^a^** | | **Weight_Den_^b^** | ***h^2^*** | **SE^b^** | **95% CI** | |  |
| **ACE models** | | | | | | | | | | | | | | | |
| ALL MDD ICD 10 | 0.4112 | 0.0060 | 0.3993 | 0.4230 | 28,078.748 | 0.3600 | 0.0082 | 0.3450 | 0.3765 | 14,852.394 | 0.3935 | 0.0048 | 0.3841 | 0.4030 | 4.68*10^-7^ |
| All MDD ICD 8, 9, 10 | 0.4205 | 0.0059 | 0.4086 | 0.4320 | 28,931.921 | 0.3596 | 0.0083 | 0.3439 | 0.3770 | 14,583.663 | 0.4001 | 0.0048 | 0.3907 | 0.4095 | 2.23*10^-9^ |
| Psychotic MDD | 0.1965 | 0.0887 | 0.0001 | 0.2804 | 127.041 | 0.2998 | 0.1589 | 0.0001 | 0.4368 | 39.613 | 0.2211 | 0.0775 | 0.0692 | 0.3729 | 5.70*10^-1^ |
| **AE models** | | | | | | | | | | | | | | | |
| ALL MDD ICD 10 | 0.4115 | 0.0060 | 0.3995 | 0.4232 | 28,176.898 | 0.3602 | 0.0082 | 0.3452 | 0.3766 | 14,877.894 | 0.3937 | 0.0048 | 0.3843 | 0.4032 | 4.44*10^-7^ |
| All MDD ICD 8, 9, 10 | 0.4208 | 0.0058 | 0.4091 | 0.4322 | 29,380.475 | 0.3598 | 0.0083 | 0.3441 | 0.3772 | 14,611.374 | 0.4005 | 0.0048 | 0.3912 | 0.4099 | 1.70*10^-9^ |
| Psychotic MDD | 0.1968 | 0.0538 | 0.0847 | 0.2882 | 345.374 | 0.3703 | 0.0435 | 0.2818 | 0.4552 | 527.823 | 0.3017 | 0.0338 | 0.2353 | 0.3680 | 1.22*10^-2^ |

^a^Estimates from Bootstrapped resampling analyses $SE=Bootstrap SD$; 95% CI = [estimate 26^th^; estimate 975^th^]

^b^Weight ^=^ $\frac{1}{{SE}^{2}}$ | ^c^SE_pooled= $\sqrt{\frac{1}{\mathrm{Weight}_{\mathrm{Swe}}+ \mathrm{Weight}_{\mathrm{Den}}}}$ (Details in eMethods 6)

^c^Post-hoc calculation to test for differences in the 2 estimates under the assumption of independency: $\Delta e={estimate}_{1}-{estimate}_{2}$; $SE_{\Delta}=\sqrt{SE_{1}^{2}+SE_{2}^{2}}$ ; z-value = $z_{\Delta}=\frac{\Delta e}{SE_{\Delta}}$; p-value = $2\cdot\Phi\left( -abs\left( z_{\Delta} \right) \right)$

**eTable 10:** Hypothesis test for difference in heritability estimates

| **Register data** | ***h^2^*** | **SE** | ***h^2^*** | **SE** | **Mean_diff** | **SE_diff** | **p-value** |
| --- | --- | --- | --- | --- | --- | --- | --- |
|  | **All MDD ICD 8, 9, 10** | | **Psychotic MDD** | |  |  |  |
| **Sweden** | 0.4208 | 0.0058 | 0.1968 | 0.0538 | 0.2207 | 0.0540 | <0.001 |
| **Denmark** | 0.3598 | 0.0083 | 0.3703 | 0.0435 | 0.0117 | 0.0435 | 0.380 |

*All estimates came from the original AE models of the whole population.*

*SE: Standard deviation of 1000 Bootstrapped estimates*

*Mean_diff: Mean of 1000 Bootstrapped differences. The diff variable was calculated as bootstrap estimates of the larger point estimate - bootstrap estimates of the smaller point estimate.*

*SE_diff: Standard deviation of 1000 Bootstrapped differences*

*p-value: Empirical one-sided p-value based on Bootstrap distribution of the difference; calculated as proportion with difference ≤ 0 out of all valid Bootstrap replicates.*

## **eTable 11:** Genetic correlation between psychotic subgroups of MDD and SCZ/SAD, BD

|  | **Sweden** | | | | | **Denmark** | | | | | **Combined estimates** | | | |  |
| --- | --- | --- | --- | --- | --- | --- | --- | --- | --- | --- | --- | --- | --- | --- | --- |
| ***r_g_* with** | ***r_g_*** | **SE** | **95% CI** | | **Weight** | ***r_g_*** | **SE** | **95% CI** | | **Weight** | ***r_g_*** | **SE** | **95% CI** | |  |
| **Schizophrenia/schizoaffective disorder** | | | | | | | | | | | | | | | |
| **Psychotic MDD** | 0.82 | 0.1140 | 0.62 | 1.00 | 76.95 | 0.62 | 0.0716 | 0.52 | 0.76 | 195.32 | 0.67 | 0.0606 | 0.55 | 0.79 |  |
| **Non-psychotic MDD** | 0.41 | 0.0191 | 0.38 | 0.46 | 2741.15 | 0.53 | 0.0228 | 0.49 | 0.57 | 1925.36 | 0.46 | 0.0146 | 0.43 | 0.49 |  |
| **Bipolar disorder** | 0.54 | 0.0271 | 0.49 | 0.61 | 1361.64 | 0.50 | 0.0369 | 0.44 | 0.56 | 734.74 | 0.53 | 0.0218 | 0.48 | 0.57 |  |
| **Bipolar disorder** | | | | | | | | | | | | | | | |
| **Psychotic MDD** | 0.69 | 0.1290 | 0.53 | 1.00 | 60.09 | 0.45 | 0.2350 | 0.15 | 1.00 | 18.10 | 0.64 | 0.1131 | 0.41 | 0.86 |  |
| **Non-psychotic MDD** | 0.72 | 0.0159 | 0.70 | 0.76 | 3955.54 | 0.62 | 0.0276 | 0.57 | 0.67 | 1316.28 | 0.70 | 0.0138 | 0.67 | 0.72 |  |
| **Non-psychotic MDD** | | | | | | | | | | | | | | | |
| **Psychotic MDD** | 0.85 | 0.0525 | 0.75 | 0.99 | 362.81 | 0.70 | 0.1150 | 0.61 | 0.99 | 75.58 | 0.82 | 0.0478 | 0.73 | 0.92 |  |
|  |  |  |  |  |  |  |  |  |  |  | Test for difference from 1^a^  p-value = 1.66*10^-4^ | | | |  |

*Estimates from AE models. Combined estimates were presented in Figure 2a. 95% CI were estimated using Bootstrap resampling with 1000 replicates. The model to estimate r_g_ between psychotic MDD and non-psychotic MDD used maximum likelihood (ML), other models used weighted least square (WLS).*

*^a^p-value for a two-tailed test to determine if the estimate is different from 1, given its standard error; p-value=2*pnorm(-abs((r_g_-1)/se))*

*MDD: Major depressive disorder | SCZ/SAD: Schizophrenia/schizoaffective disorder | BD: bipolar disorder*

## **eTable 12**: Genetic correlation between psychotic MDD, MDD, SCZ/SAD, BD and psychiatric disorders

|  | **Sweden** | | | | | **Denmark** | | | | | **Combined estimates** | | | |
| --- | --- | --- | --- | --- | --- | --- | --- | --- | --- | --- | --- | --- | --- | --- |
| ***r_g_* with** | ***r_g_*** | **SE** | **95% CI** | | **Weight** | ***r_g_*** | **SE** | **95% CI** | | **Weight** | ***r_g_*** | **SE** | **95% CI** | |
| **Psychotic MDD** | | | | | | | | | | | | | | |
| Anxiety | 0.76 | 0.1175 | 0.59 | 1.00 | 72.43 | 0.74 | 0.1693 | 0.48 | 1.00 | 34.87 | 0.75 | 0.0965 | 0.56 | 0.94 |
| OCD | 0.60 | 0.1393 | 0.40 | 1.00 | 51.53 | 0.51 | 0.2787 | 0.20 | 1.00 | 12.87 | 0.58 | 0.1246 | 0.34 | 0.83 |
| PTSD | 0.74 | 0.1414 | 0.58 | 1.00 | 50.02 | 0.53 | 0.0426 | 0.45 | 0.62 | 550.80 | 0.54 | 0.0408 | 0.46 | 0.62 |
| Substance use | 0.52 | 0.1069 | 0.39 | 0.82 | 87.51 | 0.41 | 0.0396 | 0.34 | 0.49 | 637.43 | 0.42 | 0.0371 | 0.35 | 0.49 |
| Eating disorder | 0.51 | 0.1455 | 0.29 | 0.87 | 47.24 | 0.43 | 0.2774 | 0.25 | 1.00 | 12.99 | 0.49 | 0.1289 | 0.24 | 0.74 |
| Self-harm | 0.67 | 0.1124 | 0.53 | 1.00 | 79.15 | 0.58 | 0.1003 | 0.46 | 1.00 | 99.48 | 0.62 | 0.0748 | 0.48 | 0.77 |
| ASD | 0.42 | 0.1148 | 0.26 | 0.72 | 75.88 | 0.14 | 0.5320 | -1.00 | 1.00 | 3.53 | 0.41 | 0.1122 | 0.19 | 0.63 |
| ADHD | 0.34 | 0.0876 | 0.21 | 0.55 | 130.31 | 0.47 | 0.2661 | 0.32 | 1.00 | 14.12 | 0.35 | 0.0832 | 0.19 | 0.52 |
| **All MDD ICD 8, 9, 10** | | | | | | | | | | | | | | |
| Anxiety | 0.90 | 0.0092 | 0.88 | 0.91 | 11,814.74 | 0.80 | 0.0175 | 0.76 | 0.83 | 3,262.32 | 0.88 | 0.0081 | 0.86 | 0.89 |
| OCD | 0.63 | 0.0218 | 0.59 | 0.67 | 2,104.20 | 0.53 | 0.0350 | 0.47 | 0.60 | 817.12 | 0.60 | 0.0185 | 0.57 | 0.64 |
| PTSD | 0.87 | 0.0102 | 0.85 | 0.89 | 9,611.69 | 0.82 | 0.0121 | 0.80 | 0.84 | 6,794.15 | 0.85 | 0.0078 | 0.83 | 0.86 |
| Substance use | 0.70 | 0.0083 | 0.68 | 0.71 | 14,515.89 | 0.57 | 0.0121 | 0.55 | 0.60 | 6,872.10 | 0.66 | 0.0068 | 0.65 | 0.67 |
| Eating disorder | 0.57 | 0.0241 | 0.53 | 0.63 | 1,721.73 | 0.49 | 0.0410 | 0.42 | 0.57 | 594.80 | 0.55 | 0.0208 | 0.51 | 0.59 |
| Self-harm | 0.80 | 0.0100 | 0.78 | 0.82 | 10,000.00 | 0.71 | 0.0245 | 0.66 | 0.75 | 1,660.14 | 0.79 | 0.0093 | 0.77 | 0.81 |
| ASD | 0.56 | 0.0150 | 0.53 | 0.59 | 4,444.44 | 0.31 | 0.0733 | 0.22 | 0.42 | 185.91 | 0.55 | 0.0147 | 0.52 | 0.58 |
| ADHD | 0.68 | 0.0105 | 0.66 | 0.71 | 9,070.29 | 0.62 | 0.0200 | 0.59 | 0.67 | 2,498.00 | 0.67 | 0.0093 | 0.65 | 0.69 |
| **Schizophrenia/schizoaffective disorder** | | | | | | | | | | | | | | |
| Anxiety | 0.48 | 0.0191 | 0.44 | 0.52 | 2,741.15 | 0.63 | 0.0276 | 0.58 | 0.68 | 1,316.18 | 0.53 | 0.0157 | 0.50 | 0.56 |
| OCD | 0.42 | 0.0402 | 0.34 | 0.50 | 618.80 | 0.43 | 0.0892 | 0.32 | 0.67 | 125.73 | 0.42 | 0.0366 | 0.35 | 0.49 |
| PTSD | 0.43 | 0.0237 | 0.38 | 0.47 | 1,780.34 | 0.65 | 0.0162 | 0.62 | 0.68 | 3,790.71 | 0.58 | 0.0134 | 0.55 | 0.61 |
| Substance use | 0.44 | 0.0167 | 0.41 | 0.47 | 3,585.64 | 0.57 | 0.0153 | 0.54 | 0.60 | 4,286.42 | 0.51 | 0.0113 | 0.49 | 0.54 |
| Eating disorder | 0.27 | 0.0683 | 0.16 | 0.36 | 214.37 | 0.27 | 0.0519 | 0.19 | 0.38 | 370.86 | 0.27 | 0.0413 | 0.19 | 0.35 |
| Self-harm | 0.47 | 0.0229 | 0.44 | 0.52 | 1,906.90 | 0.58 | 0.0344 | 0.52 | 0.65 | 845.20 | 0.51 | 0.0191 | 0.47 | 0.54 |
| ASD | 0.45 | 0.0330 | 0.39 | 0.52 | 918.27 | 0.17 | 0.1609 | 0.04 | 0.89 | 38.63 | 0.44 | 0.0323 | 0.38 | 0.50 |
| ADHD | 0.30 | 0.1027 | 0.24 | 0.34 | 94.81 | 0.44 | 0.0276 | 0.58 | 0.68 | 1,316.18 | 0.43 | 0.0266 | 0.38 | 0.48 |
| **Bipolar disorder** | | | | | | | | | | | | | | |
| Anxiety | 0.63 | 0.0162 | 0.61 | 0.67 | 3,810.39 | 0.41 | 0.0360 | 0.34 | 0.49 | 771.39 | 0.59 | 0.0148 | 0.56 | 0.62 |
| OCD | 0.40 | 0.0311 | 0.35 | 0.47 | 1,033.90 | 0.35 | 0.1222 | 0.14 | 0.67 | 66.92 | 0.40 | 0.0301 | 0.34 | 0.46 |
| PTSD | 0.61 | 0.0176 | 0.58 | 0.65 | 3,228.31 | 0.45 | 0.0223 | 0.40 | 0.49 | 2,016.14 | 0.55 | 0.0138 | 0.52 | 0.57 |
| Substance use | 0.53 | 0.0147 | 0.50 | 0.56 | 4,627.70 | 0.33 | 0.0203 | 0.29 | 0.37 | 2,427.37 | 0.46 | 0.0119 | 0.44 | 0.48 |
| Eating disorder | 0.43 | 0.4535 | -1.00 | 0.50 | 4.86 | 0.33 | 0.0929 | 0.14 | 0.50 | 115.96 | 0.33 | 0.0910 | 0.15 | 0.51 |
| Self-harm | 0.59 | 0.0158 | 0.57 | 0.63 | 4,005.77 | 0.43 | 0.0572 | 0.34 | 0.52 | 305.92 | 0.58 | 0.0152 | 0.55 | 0.61 |
| ASD | 0.44 | 0.0250 | 0.39 | 0.49 | 1,600.00 | 0.21 | 0.2911 | 0.02 | 1.00 | 11.80 | 0.44 | 0.0249 | 0.39 | 0.49 |
| ADHD | 0.55 | 0.0161 | 0.53 | 0.59 | 3,857.88 | 0.37 | 0.0749 | 0.31 | 0.60 | 178.47 | 0.54 | 0.0157 | 0.51 | 0.57 |

*Estimates from AE models. Combined estimates were presented in Figure 2b.*

*All estimates came from the original model of the whole population. SE: Standard deviation of 1000 Bootstrapped estimates*

*MDD: Major depressive disorder | SCZ/SAD: Schizophrenia/schizoaffective disorder | BD: bipolar disorder | OCD: Obsessive-Compulsive Disorder | PTSD: Post-Traumatic Stress Disorder | ASD: Autism Spectrum Disorder | ADHD: Attention-Deficit/Hyperactivity Disorder*

## **eTable 13**: Hypothesis testing for difference in genetic correlations

|  | | | | | | | | | |
| --- | --- | --- | --- | --- | --- | --- | --- | --- | --- |
| ***r_g_* with** | ***r_g_*** | **SE** | ***r_g_*** | **SE** | $\boldsymbol{abs(\Delta}\boldsymbol{r)}$ | $\boldsymbol{S}\boldsymbol{E}_{\boldsymbol{\Delta}}$ | | **p-value** | |
|  | **Psychotic MDD** | | **Non-psychotic MDD** | |  |  | |  | |
| Schizophrenia | 0.67 | 0.0606 | 0.46 | 0.0146 | 0.21 | 0.0623 | | **7.55*10^-4^** | |
| Bipolar disorder | 0.64 | 0.1131 | 0.70 | 0.0138 | 0.06 | 0.1139 | | 5.98*10^-1^ | |
|  | **Psychotic MDD** | | **All MDD ICD 8, 9, 10** | |  | |  | |  |
| Anxiety | 0.75 | 0.0965 | 0.88 | 0.0081 | 0.13 | | 0.0968 | | 1.79*10^-1^ |
| OCD | 0.58 | 0.1246 | 0.60 | 0.0185 | 0.02 | | 0.126 | | 8.74*10^-1^ |
| PTSD | 0.54 | 0.0408 | 0.85 | 0.0078 | 0.31 | | 0.0415 | | **8.46*10^-14^** |
| Substance use | 0.42 | 0.0371 | 0.66 | 0.0068 | 0.24 | | 0.0377 | | **1.98*10^-10^** |
| Eating disorder | 0.49 | 0.1289 | 0.55 | 0.0208 | 0.06 | | 0.1306 | | 6.46*10^-1^ |
| Self-harm | 0.62 | 0.0748 | 0.79 | 0.0093 | 0.17 | | 0.0754 | | 2.41*10^-2^ |
| ASD | 0.41 | 0.1122 | 0.55 | 0.0147 | 0.14 | | 0.1132 | | 2.16*10^-1^ |
| ADHD | 0.35 | 0.0832 | 0.67 | 0.0093 | 0.32 | | 0.0837 | | **1.32*10^-4^** |
|  | **Psychotic MDD** | | **Schizophrenia** | |  | |  | |  |
| Anxiety | 0.75 | 0.0965 | 0.53 | 0.0157 | 0.22 | | 0.0978 | | 2.44*10^-2^ |
| OCD | 0.58 | 0.1246 | 0.42 | 0.0366 | 0.16 | | 0.1299 | | 2.18*10^-1^ |
| PTSD | 0.54 | 0.0408 | 0.58 | 0.0134 | 0.04 | | 0.0429 | | 3.52*10^-1^ |
| Substance use | 0.42 | 0.0371 | 0.51 | 0.0113 | 0.09 | | 0.0388 | | 2.03*10^-2^ |
| Eating disorder | 0.49 | 0.1289 | 0.27 | 0.0413 | 0.22 | | 0.1354 | | 1.04*10^-1^ |
| Self-harm | 0.62 | 0.0748 | 0.51 | 0.0191 | 0.11 | | 0.0772 | | 1.54*10^-1^ |
| ASD | 0.41 | 0.1122 | 0.44 | 0.0323 | 0.03 | | 0.1168 | | 7.97*10^-1^ |
| ADHD | 0.35 | 0.0832 | 0.43 | 0.0266 | 0.08 | | 0.0873 | | 3.60*10^-1^ |
|  | **Psychotic MDD** | | **Bipolar disorder** | |  | |  | |  |
| Anxiety | 0.75 | 0.0965 | 0.59 | 0.0148 | 0.16 | | 0.0976 | | 1.01*10^-1^ |
| OCD | 0.58 | 0.1246 | 0.40 | 0.0301 | 0.18 | | 0.1282 | | 1.60*10^-1^ |
| PTSD | 0.54 | 0.0408 | 0.55 | 0.0138 | 0.01 | | 0.0431 | | 8.16*10^-1^ |
| Substance use | 0.42 | 0.0371 | 0.46 | 0.0119 | 0.04 | | 0.039 | | 3.05*10^-1^ |
| Eating disorder | 0.49 | 0.1289 | 0.33 | 0.0910 | 0.16 | | 0.1578 | | 3.11*10^-1^ |
| Self-harm | 0.62 | 0.0748 | 0.58 | 0.0152 | 0.04 | | 0.0763 | | 6.00*10^-1^ |
| ASD | 0.41 | 0.1122 | 0.44 | 0.0249 | 0.03 | | 0.1149 | | 7.94*10^-1^ |
| ADHD | 0.35 | 0.0832 | 0.54 | 0.0157 | 0.19 | | 0.0847 | | 2.48*10^-2^ |

^a^Compare the combined estimates using post-hoc calculation to test for differences in the 2 *r_g_* estimates

$$\Delta r=r_{g}1-r_{g}2$$

$$SE_{\Delta}=\sqrt{SE_{r_{g}1}^{2}+SE_{r_{g}2}^{2}}$$

z-value = $z_{\Delta}=\frac{\Delta r}{SE_{\Delta}}$

p-value = $2\cdot\Phi\left( -abs\left( z_{\Delta} \right) \right)$

**Bold p-value** indicate statistical significance, p-value ≤ Bonferroni corrected p-value (0.05/26=1.92*10^-3^)

## **eTable 14:** PRS regression models separately for UKB and PREFECT

1. In UK-Biobank data, compare psychotic MDD with **all non-psychotic** MDD

| **PRS trait** | **PREFECT** | | | | **UKB** | | | | **Pooled estimate** | | |
| --- | --- | --- | --- | --- | --- | --- | --- | --- | --- | --- | --- |
|  | **OR** | **95% CI** | | **p-value** | **OR** | **95% CI** | | **p-value** | **OR** | **95% CI** | |
|  | 746 psychotic/2146 non-psychotic | | | | 542 psychotic/**25851 non-psychotic** | | | |  | | |
| **Uni-PRS models^a^** | | | | | | | | | | | |
| **MDD** | 0.93 | 0.86 | 1.02 | 1.06*10^-1^ | 0.94 | 0.86 | 1.02 | 1.29*10^-1^ | 0.93 | 0.88 | 0.99 |
| **SCZ** | 1.11 | 1.02 | 1.22 | 1.50*10^-2^ | 1.51 | 1.38 | 1.64 | 5.23*10^-21^ | 1.30 | 1.22 | 1.38 |
| **BD all types** | 1.07 | 0.98 | 1.16 | 1.48*10^-1^ | 1.53 | 1.40 | 1.67 | 3.82*10^-22^ | 1.28 | 1.20 | 1.36 |
| **BD type-I** | 1.12 | 1.03 | 1.22 | 9.50*10^-3^ | 1.58 | 1.45 | 1.73 | 2.46*10^-25^ | 1.33 | 1.25 | 1.41 |
| **BD type-II** | 0.93 | 0.85 | 1.01 | 7.70*10^-2^ | 1.09 | 1.00 | 1.19 | 4.91*10^-2^ | 1.00 | 0.95 | 1.07 |
| **Multi-PRS models^b^** | | | | | | | | | | | |
| **MDD** | 0.91 | 0.84 | 1.00 | 4.17*10^-2^ | 0.83 | 0.76 | 0.91 | 3.11*10^-5^ | 0.87 | 0.82 | 0.93 |
| **SCZ** | 1.11 | 1.01 | 1.22 | 3.07*10^-2^ | 1.36 | 1.24 | 1.49 | 1.18*10^-10^ | 1.23 | 1.15 | 1.32 |
| **BD all types** | 1.04 | 0.94 | 1.14 | 4.37*10^-1^ | 1.41 | 1.28 | 1.55 | 1.08*10^-13^ | 1.21 | 1.13 | 1.29 |
| **BD type-I** | 1.10 | 1.00 | 1.21 | 4.59*10^-2^ | 1.45 | 1.32 | 1.60 | 4.69*10^-15^ | 1.26 | 1.18 | 1.35 |
| **BD type-II** | 0.92 | 0.84 | 1.00 | 4.99*10^-2^ | 1.03 | 0.94 | 1.12 | 5.39*10^-1^ | 0.97 | 0.91 | 1.03 |

1. In UK-Biobank data, compare psychotic MDD with **severe non-psychotic** MDD

| **PRS trait** | **PREFECT** | | | | **UKB** | | | | **Pooled estimate** | | |
| --- | --- | --- | --- | --- | --- | --- | --- | --- | --- | --- | --- |
|  | **OR** | **95% CI** | | **p-value** | **OR** | **95% CI** | | **p-value** | **OR** | **95% CI** | |
|  | 746 psychotic/2146 non-psychotic | | | | 542 psychotic/**536** **severe non-psychotic** | | | |  | | |
| **Uni-PRS models^a^** | | | | | | | | | | | |
| **MDD** | 0.93 | 0.86 | 1.02 | 1.06*10^-1^ | 0.93 | 0.82 | 1.05 | 2.15*10^-1^ | 0.93 | 0.87 | 1.00 |
| **SCZ** | 1.11 | 1.02 | 1.22 | 1.50*10^-2^ | 1.41 | 1.24 | 1.60 | 1.41*10^-7^ | 1.20 | 1.12 | 1.29 |
| **BD all types** | 1.07 | 0.98 | 1.16 | 1.48*10^-1^ | 1.27 | 1.12 | 1.43 | 1.17*10^-4^ | 1.13 | 1.05 | 1.21 |
| **BD type-I** | 1.12 | 1.03 | 1.22 | 9.50*10^-3^ | 1.27 | 1.13 | 1.43 | 7.21*10^-5^ | 1.17 | 1.09 | 1.25 |
| **BD type-II** | 0.93 | 0.85 | 1.01 | 7.70*10^-2^ | 1.04 | 0.92 | 1.17 | 5.51*10^-1^ | 0.96 | 0.90 | 1.03 |
| **Multi-PRS models^b^** | | | | | | | | | | | |
| **MDD** | 0.91 | 0.84 | 1.00 | 4.17*10^-2^ | 0.85 | 0.75 | 0.97 | 1.33*10^-2^ | 0.89 | 0.83 | 0.96 |
| **SCZ** | 1.11 | 1.01 | 1.22 | 3.07*10^-2^ | 1.36 | 1.18 | 1.56 | 1.11*10^-5^ | 1.19 | 1.10 | 1.28 |
| **BD all types** | 1.04 | 0.94 | 1.14 | 4.37*10^-1^ | 1.18 | 1.03 | 1.35 | 1.39*10^-2^ | 1.08 | 1.00 | 1.17 |
| **BD type-I** | 1.10 | 1.00 | 1.21 | 4.59*10^-2^ | 1.17 | 1.03 | 1.33 | 1.33*10^-2^ | 1.12 | 1.04 | 1.21 |
| **BD type-II** | 0.92 | 0.84 | 1.00 | 4.99*10^-2^ | 1.00 | 0.88 | 1.14 | 9.90*10^-1^ | 0.94 | 0.88 | 1.01 |

*Models comparing psychotic vs non-psychotic MDD. All models included the first 5 genomic PCs as covariates*

*^a^For each definition of psychotic MDD in each dataset, we fitted 5 models:*

*(1) Psychotic MDD ~ MDD PRS + 5 PCs + sex + age; (2) Psychotic MDD ~ SCZ PRS + 5 PCs + sex + age; (3) Psychotic MDD ~ BD all types PRS + 5 PCs + sex + age; (4) Psychotic MDD ~ BD type-I PRS + 5 PCs + sex + age; (5) Psychotic MDD ~ BD type-II PRS + 5 PCs + sex + age*

*^b^For each definition of psychotic MDD in each dataset, we fitted 3 models:*

*(1) Psychotic MDD ~ MDD PRS + SCZ PRS + BD all types PRS + 5 PCs + sex + age; (2) Psychotic MDD ~ MDD PRS + SCZ PRS + BD type-I PRS + 5 PCs + sex + age; (3) Psychotic MDD ~ MDD PRS + SCZ PRS + BD type-II PRS + 5 PCs + sex + age*

## **eTable 15**: Logistic regression comparing MDD PRS between psychotic/non-psychotic MDD with non-MDD

| **Comparison** | **Uni-PRS models** | | | | **Multi-PRS models^a^** | | | | |
| --- | --- | --- | --- | --- | --- | --- | --- | --- | --- |
|  | **OR** | **95% CI** | | **p-value** | **OR** | **95% CI** | | | **p-value** |
| **Psychotic vs non-MDD** | 1.16 | 1.06 | 1.26 | 7.20*10^-4^ | 0.99 | 0.91 | 1.09 | 9.03*10^-1^ | |
| **Non-psychotic vs non-MDD** | 1.24 | 1.22 | 1.25 | 3.15*10^-240^ | 1.21 | 1.19 | 1.22 | 5.01*10^-176^ | |
| **Psychotic vs non-psychotic** | 0.94 | 0.86 | 1.02 | 1.29*10^-1^ | 0.83 | 0.76 | 0.91 | 3.11*10^-5^ | |

*Results from using UK-Biobank data, data for non-MDD group from PREFECT was not available for this project. Non-MDD was defined as not having major depression based on 7 criteria including (1) seeking help for mental health, (2) using antidepressant, (3) lifetime MDD based on Composite International Diagnostic Interview (CIDI) Short Form, (4) ICD-coded MDD, (5) Probable major depression, (6) Self-reported major depression, and (7) Cardinal symptoms of anhedonia and dysphoria. Details were published elsewhere.^24^*

*N psychotic MDD = 542*

*N non-psychotic MDD = 25851*

*N non-MDD = 433138*

*All models included the first 5 genomic PCs, sex, age as covariates*

*^a^Further adjusted for PRS of schizophrenia, bipolar disorders*

1. **Supplementary results from sensitivity analyses**

We provide results for 1) heritability, 2) genetic correlation between psychotic subgroups of MDD and schizophrenia/schizoaffective disorder, bipolar disorder using Swedish data and 3) PRS regression from sensitivity analyses for the restrictive definition of psychotic MDD

## **eTable 16**: Number of cases, concordant/discordant pairs, intraclass correlations for restrictive psychotic MDD

1. **Number of cases**

|  | **Swedish register** | **PREFECT** | **UK-Biobank** |
| --- | --- | --- | --- |
| **Restrictive Psychotic MDD** (% in all MDD^a^) | 4364 (2.52%) | 480 (26.12%) | 389 (1.53%) |

1. **Number concordant/discordant pairs**

|  |  | **Sibling/cousin 2** | | | |
| --- | --- | --- | --- | --- | --- |
|  | **Sibling/cousin 1** | **Full-sibling** | | **Cousin** | |
| **Restrictive Psychotic MDD** |  | **No** | **Yes** | **No** | **Yes** |
| No |  | 1938658 | 2325 | 5645714 | 6396 |
| Yes |  | 2382 | 9 | 6274 | 10 |

1. **Intraclass correlation**

| **Full-sibling** | | | **Cousin** | | |
| --- | --- | --- | --- | --- | --- |
| **Correlation** | **SE** | **95% CI** | **Correlation** | **SE** | **95% CI** |
| 0.115 | 0.037 | 0.043; 0.186 | 0.031 | 0.030 | -0.027; 0.090 |

*^a^Proportion among all MDD based on ICD 10 codes*

## **eTable 17:** Heritability estimates for restrictive definition of psychotic MDD

|  | ***h^2^*** | **SE^a^** | **95% CI^a^** | |
| --- | --- | --- | --- | --- |
| **ACE model** | 0.2290 | 0.1089 | 0.0001 | 0.3329 |
| **AE model** | 0.2292 | 0.0737 | 0.0753 | 0.3558 |

## **eTable 18:** Genetic correlation between psychotic subgroups of MDD based on **restrictive definition** and SCZ/SAD, BD

| ***r_g_* with** | ***r_g_*** | **SE** | **95% CI** | |
| --- | --- | --- | --- | --- |
|  | **Schizophrenia** | | | |
| **Restrictive psychotic MDD** | 0.62 | 0.1586 | 0.35 | 0.97 |
| **Restrictive Non-psychotic MDD** | 0.39 | 0.0254 | 0.34 | 0.44 |
| **Bipolar disorder** | 0.54 | 0.0271 | 0.49 | 0.61 |
|  | **Bipolar disorder** | | | |
| **Restrictive psychotic MDD** | 0.37 | 0.2852 | 0.14 | 1.00 |
| **Restrictive Non-psychotic MDD** | 0.45 | 0.0556 | 0.40 | 0.51 |
|  | **Restrictive Non-psychotic MDD** | | | |
| **Restrictive psychotic MDD** | 0.67 | 0.1137 | 0.59 | 0.99 |

*Estimates from AE models. SE and 95% CI were estimated using Bootstrap resampling with successfully optimized models, (i.e., Mx exit code 0) among the 1000 replicates. The model to estimate r_g_ between restrictive non-psychotic MDD and SCZ/SAD, and between SCZ/SAD and BD used weighted least square (WLS), other models were fitted using maximum likelihood (ML). MDD: Major depressive disorder | SCZ/SAD: Schizophrenia/schizoaffective disorder | BD: bipolar disorder*

## **eTable 19:** PRS regression models separately for UKB and PREFECT for **restrictive psychotic MDD**

1. In UK-Biobank data, compare psychotic MDD with **all non-psychotic** MDD

| **PRS trait** | **PREFECT** | | | | **UK-Biobank** | | | | | **Pooled estimate** | | |
| --- | --- | --- | --- | --- | --- | --- | --- | --- | --- | --- | --- | --- |
|  | **OR** | **95% CI** | | **p-value** | **OR** | **95% CI** | | | **p-value** | **OR** | **95% CI** | |
|  | 480 restrictive psychotic/  1356 restrictive non-psychotic | | | | 389 restrictive psychotic/ **25086 restrictive non-psychotic** | | | | |  | | |
| **Uni-PRS models^a^** | | | | | | | | | | | | |
| **MDD** | 0.95 | 0.86 | 1.06 | 3.97*10^-1^ | 0.96 | 0.87 | | 1.07 | 4.72*10^-1^ | 0.96 | 0.89 | 1.03 |
| **SCZ** | 1.14 | 1.02 | 1.27 | 2.27*10^-2^ | 1.53 | 1.38 | | 1.69 | 2.43*10^-16^ | 1.34 | 1.24 | 1.44 |
| **BD all types** | 1.13 | 1.01 | 1.26 | 3.10*10^-2^ | 1.57 | 1.42 | | 1.74 | 4.36*10^-18^ | 1.35 | 1.25 | 1.45 |
| **BD type-I** | 1.21 | 1.09 | 1.35 | 6.41*10^-4^ | 1.62 | 1.46 | | 1.80 | 3.65*10^-20^ | 1.42 | 1.31 | 1.53 |
| **BD type-II** | 0.93 | 0.83 | 1.03 | 1.58*10^-1^ | 1.12 | 1.02 | | 1.24 | 2.32*10^-2^ | 1.03 | 0.95 | 1.10 |
| **Multi-PRS models^b^** | | | | | | | | | | | | |
| **MDD** | 0.93 | 0.83 | 1.04 | 1.83*10^-1^ | 0.85 | 0.77 | 0.94 | | 2.23*10^-3^ | 0.89 | 0.82 | 0.96 |
| **SCZ** | 1.11 | 0.98 | 1.25 | 1.01*10^-1^ | 1.36 | 1.22 | 1.52 | | 4.23*10^-8^ | 1.24 | 1.14 | 1.34 |
| **BD all types** | 1.10 | 0.97 | 1.24 | 1.23*10^-1^ | 1.44 | 1.29 | 1.61 | | 1.88*10^-10^ | 1.27 | 1.17 | 1.38 |
| **BD type-I** | 1.19 | 1.06 | 1.34 | 4.04*10^-3^ | 1.48 | 1.33 | 1.66 | | 4.07*10^-12^ | 1.34 | 1.23 | 1.45 |
| **BD type-II** | 0.91 | 0.81 | 1.02 | 9.43*10^-2^ | 1.06 | 0.95 | 1.17 | | 2.96*10^-1^ | 0.99 | 0.91 | 1.06 |

1. In UK-Biobank data, compare psychotic MDD with **severe non-psychotic** MDD

| **PRS trait** | **PREFECT** | | | | **UK-Biobank** | | | | | **Pooled estimate** | | |
| --- | --- | --- | --- | --- | --- | --- | --- | --- | --- | --- | --- | --- |
|  | **OR** | **95% CI** | | **p-value** | **OR** | **95% CI** | | | **p-value** | **OR** | **95% CI** | |
|  | 480 restrictive psychotic/  1356 restrictive non-psychotic | | | | 389 restrictive psychotic/ **515 restrictive severe non-psychotic** | | | | |  | | |
| **Uni-PRS models^a^** | | | | | | | | | | | | |
| **MDD** | 0.95 | 0.86 | 1.06 | 3.9*10^-1^ | 0.94 | 0.82 | | 1.07 | 3.38*10^-1^ | 0.95 | 0.87 | 1.03 |
| **SCZ** | 1.14 | 1.02 | 1.27 | 2.27*10^-2^ | 1.38 | 1.20 | | 1.59 | 6.33*10^-6^ | 1.23 | 1.12 | 1.34 |
| **BD all types** | 1.13 | 1.01 | 1.26 | 3.10*10^-2^ | 1.36 | 1.19 | | 1.56 | 1.29*10^-5^ | 1.21 | 1.11 | 1.32 |
| **BD type-I** | 1.21 | 1.09 | 1.35 | 6.41*10^-4^ | 1.37 | 1.19 | | 1.57 | 5.71*10^-6^ | 1.27 | 1.17 | 1.39 |
| **BD type-II** | 0.93 | 0.83 | 1.03 | 1.58*10^-1^ | 1.07 | 0.94 | | 1.23 | 3.09*10^-1^ | 0.98 | 0.90 | 1.07 |
| **Multi-PRS models^b^** | | | | | | | | | | | | |
| **MDD** | 0.93 | 0.83 | 1.04 | 1.83*10^-1^ | 0.85 | 0.74 | 0.98 | | 2.87*10^-2^ | 0.90 | 0.83 | 0.98 |
| **SCZ** | 1.11 | 0.98 | 1.25 | 1.01*10^-1^ | 1.29 | 1.11 | 1.50 | | 8.00*10^-4^ | 1.18 | 1.07 | 1.29 |
| **BD all types** | 1.10 | 0.97 | 1.24 | 1.23*10^-1^ | 1.28 | 1.10 | 1.49 | | 1.16*10^-3^ | 1.17 | 1.06 | 1.28 |
| **BD type-I** | 1.19 | 1.06 | 1.34 | 4.04*10^-3^ | 1.28 | 1.11 | 1.48 | | 7.27*10^-4^ | 1.23 | 1.12 | 1.35 |
| **BD type-II** | 0.91 | 0.81 | 1.02 | 9.43*10^-2^ | 1.05 | 0.91 | 1.21 | | 5.34*10^-1^ | 0.96 | 0.88 | 1.05 |

*Models comparing psychotic vs non-psychotic MDD. All models included the first 5 genomic PCs as covariates*

*^a^We fitted 5 models: 1) Psychotic MDD ~ MDD PRS + 5 PCs + sex + age; 2) Psychotic MDD ~ SCZ PRS + 5 PCs + sex + age; 3) Psychotic MDD ~ BD all types PRS + 5 PCs + sex + age; 4) Psychotic MDD ~ BD type-I PRS + 5 PCs + sex + age; 5) Psychotic MDD ~ BD type-II PRS + 5 PCs + sex + age*

*^b^We fitted 3 models: 1) Psychotic MDD ~ MDD PRS + SCZ PRS + BD all types PRS + 5 PCs + sex + age; 2) Psychotic MDD ~ MDD PRS + SCZ PRS + BD type-I PRS + 5 PCs + sex + age; 3) Psychotic MDD ~ MDD PRS + SCZ PRS + BD type-II PRS + 5 PCs + sex + age*

# **References**

1. Ludvigsson JF, Andersson E, Ekbom A, et al. External review and validation of the Swedish national inpatient register. *BMC Public Health.* 2011;11(1):450.

2. Schmidt M, Schmidt SA, Sandegaard JL, Ehrenstein V, Pedersen L, Sørensen HT. The Danish National Patient Registry: a review of content, data quality, and research potential. *Clin Epidemiol.* 2015;7:449-490.

3. Brooke HL, Talbäck M, Hörnblad J, et al. The Swedish cause of death register. *Eur J Epidemiol.* 2017;32(9):765-773.

4. Helweg-Larsen K. The Danish Register of Causes of Death. *Scand J Public Health.* 2011;39(7 Suppl):26-29.

5. Ludvigsson JF, Almqvist C, Bonamy AK, et al. Registers of the Swedish total population and their use in medical research. *Eur J Epidemiol.* 2016;31(2):125-136.

6. Schmidt M, Pedersen L, Sørensen HT. The Danish Civil Registration System as a tool in epidemiology. *European Journal of Epidemiology.* 2014;29(8):541-549.

7. Ekbom A. The Swedish Multi-generation Register. *Methods Mol Biol.* 2011;675:215-220.

8. Due JKE. *Hvidbog for et dansk multigenerationsregister: - en infrastruktur for fremtidens forskning.* Det Koordinerende Organ for Registerforskning; 2018.

9. Wettermark B, Hammar N, MichaelFored C, et al. The new Swedish Prescribed Drug Register—Opportunities for pharmacoepidemiological research and experience from the first six months. *Pharmacoepidemiology and Drug Safety.* 2007;16(7):726-735.

10. Bycroft C, Freeman C, Petkova D, et al. The UK Biobank resource with deep phenotyping and genomic data. *Nature.* 2018;562(7726):203-209.

11. Clements CC, Karlsson R, Lu Y, et al. Genome-wide association study of patients with a severe major depressive episode treated with electroconvulsive therapy. *Molecular Psychiatry.* 2021;26(6):2429-2439.

12. Galinsky KJ, Bhatia G, Loh PR, et al. Fast Principal-Component Analysis Reveals Convergent Evolution of ADH1B in Europe and East Asia. *Am J Hum Genet.* 2016;98(3):456-472.

13. Price AL, Patterson NJ, Plenge RM, Weinblatt ME, Shadick NA, Reich D. Principal components analysis corrects for stratification in genome-wide association studies. *Nat Genet.* 2006;38(8):904-909.

14. Zetterqvist J, Sjölander A. Doubly Robust Estimation with the R Package drgee. 2015;4(1):69-86.

15. Boker S, Neale M, Maes H, et al. OpenMx: An Open Source Extended Structural Equation Modeling Framework. *Psychometrika.* 2011;76(2):306-317.

16. Nguyen TD, Kowalec K, Pasman J, et al. Genetic Contribution to the Heterogeneity of Major Depressive Disorder: Evidence From a Sibling-Based Design Using Swedish National Registers. *Am J Psychiatry.* 2023;180(10):714-722.

17. Wray NR, Ripke S, Mattheisen M, et al. Genome-wide association analyses identify 44 risk variants and refine the genetic architecture of major depression. *Nat Genet.* 2018;50(5):668-681.

18. Trubetskoy V, Pardiñas AF, Qi T, et al. Mapping genomic loci implicates genes and synaptic biology in schizophrenia. *Nature.* 2022;604(7906):502-508.

19. Mullins N, Forstner AJ, O'Connell KS, et al. Genome-wide association study of more than 40,000 bipolar disorder cases provides new insights into the underlying biology. *Nat Genet.* 2021;53(6):817-829.

20. Lloyd-Jones LR, Zeng J, Sidorenko J, et al. Improved polygenic prediction by Bayesian multiple regression on summary statistics. *Nature Communications.* 2019;10(1):5086.

21. Chang CC, Chow CC, Tellier LCAM, Vattikuti S, Purcell SM, Lee JJ. Second-generation PLINK: rising to the challenge of larger and richer datasets. *GigaScience.* 2015;4(1):s13742-13015-10047-13748.

22. Fixed-Effect Versus Random-Effects Models. In: *Introduction to Meta‐Analysis.*2009:77-86.

23. Kendler KS, Ohlsson H, Sundquist J, Sundquist K. Family Genetic Risk Scores and the Genetic Architecture of Major Affective and Psychotic Disorders in a Swedish National Sample. *JAMA Psychiatry.* 2021;78(7):735-743.

24. Nguyen TD, Harder A, Xiong Y, et al. Genetic heterogeneity and subtypes of major depression. *Mol Psychiatry.* 2022;27(3):1667-1675.
